# A generalized deep learning framework for whole-slide image segmentation and analysis

**DOI:** 10.1038/s41598-021-90444-8

**Published:** 2021-06-02

**Authors:** Mahendra Khened, Avinash Kori, Haran Rajkumar, Ganapathy Krishnamurthi, Balaji Srinivasan

**Affiliations:** 1 Department of Engineering Design, Indian Institute of Technology Madras, Chennai, 600036 India; 2 Department of Mechanical Engineering, Indian Institute of Technology Madras, Chennai, 600036 India

**Keywords:** Medical imaging, Machine learning, Software

## Abstract

Histopathology tissue analysis is considered the gold standard in cancer diagnosis and prognosis. Whole-slide imaging (WSI), i.e., the scanning and digitization of entire histology slides, are now being adopted across the world in pathology labs. Trained histopathologists can provide an accurate diagnosis of biopsy specimens based on WSI data. Given the dimensionality of WSIs and the increase in the number of potential cancer cases, analyzing these images is a time-consuming process. Automated segmentation of tumorous tissue helps in elevating the precision, speed, and reproducibility of research. In the recent past, deep learning-based techniques have provided state-of-the-art results in a wide variety of image analysis tasks, including the analysis of digitized slides. However, deep learning-based solutions pose many technical challenges, including the large size of WSI data, heterogeneity in images, and complexity of features. In this study, we propose a generalized deep learning-based framework for histopathology tissue analysis to address these challenges. Our framework is, in essence, a sequence of individual techniques in the preprocessing-training-inference pipeline which, in conjunction, improve the efficiency and the generalizability of the analysis. The combination of techniques we have introduced includes an ensemble segmentation model, division of the WSI into smaller overlapping patches while addressing class imbalances, efficient techniques for inference, and an efficient, patch-based uncertainty estimation framework. Our ensemble consists of DenseNet-121, Inception-ResNet-V2, and DeeplabV3Plus, where all the networks were trained end to end for every task. We demonstrate the efficacy and improved generalizability of our framework by evaluating it on a variety of histopathology tasks including breast cancer metastases (CAMELYON), colon cancer (DigestPath), and liver cancer (PAIP). Our proposed framework has state-of-the-art performance across all these tasks and is ranked within the top 5 currently for the challenges based on these datasets. The entire framework along with the trained models and the related documentation are made freely available at GitHub and PyPi. Our framework is expected to aid histopathologists in accurate and efficient initial diagnosis. Moreover, the estimated uncertainty maps will help clinicians to make informed decisions and further treatment planning or analysis.

## Introduction

Histopathology is considered the gold standard for cancer diagnosis^1,2^ and identification of prognostic and therapeutic targets. Early diagnosis of cancer significantly increases the probability of survival^3^. Unfortunately, pathological analysis is an arduous process that is difficult, time-consuming, and requires in-depth knowledge. A study^4^ examining breast biopsies concordance among pathologists found that pathologists disagreed with each other on a diagnosis 24.7% of the time on average. This high rate of misdiagnosis stresses the need to develop computer-aided methods to aid pathologists in histopathology.

Digital pathology is the method of digitizing the histology slides to produce high-resolution images^5^. Studies have been conducted on collecting, analyzing, and interpreting digitized pathological slide images^1^. The increasing prevalence of WSI technology that can scan the entire tissue slide at the subcellular level makes the in-silico pathology analysis more viable^6^. Digital pathology’s array of image analysis activities include identification and counting (e.g. mitotic events), segmentation (e.g. nuclei), and tissue differentiation (e.g. cancerous vs. non-cancerous)^5,7,8^. Segmentation analysis helps to detect and separate tumor cells from the normal cells^9,10^. Segmentation of WSI images is usually the precursor for performing various other downstream analyses such as classification and tumor burden estimation.

Automated WSI image analysis is plagued by a myriad of challenges^11^, namely:

1. *Large dimensionality of WSI images* A WSI image is obtained by digitizing a glass slide at a very high resolution (of the order of 0.25 micrometers/pixel, which corresponds to 40 $\times$ magnification on a microscope). A typical glass-slide of size 20 mm $\times$ 15 mm results in gigapixel image of size 80,000 $\times$ 60,000 pixels.
2. *Insufficient training samples* The main impediments to the development and clinical implementation of deep learning algorithms consist of sufficiently large, curated, and representative training data which includes expert labeling, which is a costly and time-consuming process (e.g., pathologist annotated data). Most clinical research groups currently have restricted access to data. The data is often based on small sample sizes with limited geographic variety, resulting in algorithms with limited utility and poor generalization.
3. *Stain variability across laboratories* As the data is acquired from multiple sources, there exists a lack of uniformity in staining protocol. Building a generalized framework that is invariant to stain pattern variability is challenging.
4. *Extraction of clinically relevant features and information* Another major challenge is trying to extract features that are meaningful from a clinical point of view. Deep learning does an excellent task of automatic feature extraction, but understanding these extracted features and extracting meaningful information from them is challenging.

In this study, we propose a generalized deep learning-based framework for histopathology tissue analysis that addresses all the aforementioned problems. Our proposed framework is a sequence of individual techniques in the preprocessing-training-inference pipeline which, in conjunction, improve the efficiency and the generalizability of the histopathology image analysis. The combination of techniques we have introduced includes an ensemble segmentation model, division of the WSI into smaller overlapping patches while addressing class imbalances, efficient techniques for inference, and an efficient, patch-based uncertainty estimation framework. The organization of the paper is as follows. Prior work on histopathology image analysis using deep learning-based methods is discussed in “Related work” section. In “Datasets used for this study” section, the datasets used in this study are presented. Discussion on training and inference pipelines is provided in “Training pipeline” and “Inference pipeline” sections respectively. Experimental analysis is described in the supplementary note. The comprehensive results to demonstrate the performance of the proposed method on several open-source datasets are presented in “Challenge results” section. Discussion of the results, conclusion, and limitations of the proposed study with the possible course of research is provided in “Discussion and conclusions” section.

## Related work

### Deep learning methods for histopathological image analysis

The advent of WSI scanners has enabled the digitization of glass slides at very high resolution. Typical WSI images are in the order of gigapixels and are usually stored in multi-resolution pyramidal format. These slide images are suitable for developing computer-aided diagnosis systems for automating the pathologist workflow. The availability of a large amount of data makes them amenable for analysis with machine learning algorithms.

In tumor pathology, nuclear morphology and cellular anatomy are often significant determinants of disease severity. In order to make the diagnostic and grading task of tumors less subjective, quantifiable features are derived from the images that correlate with the condition of the disease^1^. For example, algorithms can be designed to detect invasive tumors by first segmenting nuclei from the background, quantifying several nuclear characteristics, such as size, shape, and distribution, and comparing these characteristics with those of normal cells^12^. Yu et al.^13^ predicted non-small cell lung cancer prognosis by applying classical machine learning algorithms that use engineered features derived from pathology images.

Feature-engineered algorithms rely on a predetermined set of features to classify the tissue. They can only classify the tissue as good as the features that differentiate between them. Thus, there is a limit to their efficiency even when there is a large amount of data available to refine the algorithm. Therefore, there has been a significant shift in recent years towards applying deep learning algorithms as they are known for their inherent ability to automatically derive features from input data. Typical deep learning-based approaches for WSI image segmentation or classification are usually made by cropping the slide image into multiple small image patches and treating them independently during training and inference. Furthermore, to make an overall slide-level prediction or generate a heatmap of regions of interest, patch-level predictions are aggregated appropriately. Cruz-Roa et al.^14^ were one of the first to use this method and showed promising results in detecting invasive ductal breast carcinoma. Several studies have applied deep learning algorithms for various pathology tasks related to breast cancer, prostate biopsies, colon cancer, etc.

Given the image size and resolution of WSI images, a patch-based approach is used for training deep convolutional neural networks^15–19^. Hameed et al.^20^ and Li et al.^21^ proposed an ensemble-based framework for classification and segmentation using histopathology images. Qin et al.^22^ proposed a feature pyramid-based approach for semantic segmentation, authors combined feature pyramid blocks in the decoder blocks along with ResNet50 as an encoder. Authors claim that including feature pyramid block provided an overall boost of 10–20% dice coefficient, with an overall dice coefficient of 63%. Guo et al.^15^ proposed a two-stage approach where the first stage utilizes inception-v3 for classifying the tumor region followed by a cascaded deep convolutional network for fine segmentation. Pedersen et al.^23^ proposed a C++-based open-source package to read, visualize, zoom, pan, and analyze WSI images using CNN’s. In another interesting study, Shahidi^24^ proposed the method to use super-resolution generative adversarial networks to generate histopathology images, they tested their approach on CAMELYON16 dataset^25^. Priego-Torres et al.^17^ propose a segmentation pipeline for breast cancer images, using a patch-based approach, where the patches we extracted from all possible regions in an image and later merged with fully connected conditional random fields (CRF). Roy et al.^19^presented a multi-resolution-based deep learning approach along with customized reconstruction loss for viable tumor segmentation in liver WSI images. Hägele et al.^26^ explored the effect of various biases in histopathology image analysis. The authors provided an explainable method such as layer-wise relevance propagation to analyze latent biases and observed and improved area under receiver operating curve by 5% after reducing a labeling bias.

Colorectal carcinoma is the third most common cancer in the world^27^. The majority of colorectal carcinoma are adenocarcinomas originating from epithelial cells^28^. Shapcott^29^ discuss the application of deep learning methods for cell identification on TCGA data. Kather et al.^30^ discuss the deep learning methods to predict the clinical course of colorectal cancer patients based on histopathological images. Bychkov et al.^31^ discuss the use of Long short-term memory (LSTM)^32^ artificial recurrent neural network (RNN) architecture for estimating the patient risk score using spatial, sequential memory.

A review on WSI application for histopathological analysis of liver diseases and for understanding liver biology is given by Melo et al.^33^. They explore how WSI can enhance the evaluation and quantification of several histologic hepatic parameters and help to identify various liver diseases with clinical implications. Kiani et al.^34^ developed a deep learning-based system to aid pathologists in differentiating between two subtypes of primary liver cancer, hepatocellular carcinoma, and cholangiocarcinoma on H&E stained WSI images. Lu and Daigle^35^ demonstrated the usefulness of extracting image features from hepatocellular carcinoma histopathological images using pre-trained CNN models to reliably differentiate between normal and cancer samples.

### Contributions

A deep learning-based framework for the segmentation and analysis of WSI images has been proposed. The framework comprises a segmentation network at its core along with novel algorithms that utilize the segmentation to do pathological analyses such as metastasis classification and viable tumor burden estimation. As discussed in “Introduction” section, challenges in WSI image analysis are mainly due to their large size, variability in staining, and the limited amount of annotated data. Although there exist some studies as described in “Related work” section, none of them seem to provide a framework that generalizes well against multiple cancer sites. In this work, we proposed methods to address most of the challenges associated with WSI analysis with the generic framework, which produces benchmarking results along with uncertainty maps on three large open-source databases. The main contributions in this work are described below :

- *Ensemble segmentation model* The ensemble comprises multiple fully convolutional architectures (FCN) architectures, each independently trained on different subsets of the training data. During inference, the ensemble generated the tumor probability map by averaging the posterior probability maps of all the FCNs. The ensemble approach showed superior segmentation performance when compared to its individual constituting FCNs.
- *Training pipeline* The proposed approach divided the WSIs into smaller-sized image patches for the purpose of training FCN models. For the preparation of the training set, efficient methodologies for sampling patches from the WSI images were introduced. The problem of class imbalance due to the limited number of representative patches from tumor regions in the WSI images was addressed by employing overlapping and oversampling techniques during patch extraction (random patch coordinate perturbation technique) alongside various data augmentation schemes.
- *Inference pipeline* For efficiently generating model inference on the entire WSI image, a concept of generating patch coordinate sampling grid from the post-processed tissue mask was introduced. The sampling grid aided in the reduction of computational time by discarding non-tissue patches during the construction of the tumor probability heatmap. The patch-based segmentation of WSI images introduced edge artifacts due to loss of neighboring context information at patch borders, and this issue was addressed by proposing techniques to average prediction probabilities at overlapping regions and making use of large patch size during inference. Apart from this, we also compute inference on multiple models parallelly for ensemble calculation over patches rather than an entire image.
- *Lymph node metastases classification from WSI images* A Random Forest-based ensemble classification algorithm was trained with hand-crafted features derived from the predicted tumor-probability maps. The class imbalance in the training dataset was addressed by employing strategies such as over-sampling (by synthetically generating under-presented class data points) and under-sampling (balance all classes by removing some noisy data points).
- *Uncertainty estimation* An efficient patch-based uncertainty estimation framework was developed to estimate both data specific and model (parameter) specific uncertainties.
- Open-source Packaging: The proposed framework was packaged into an open-source GUI application for the benefit of researchers (DigiPathAI on GitHub).

The performance of the segmentation pipeline was benchmarked by validating it on WSI slide images of three different cancer sites, namely- breast lymph nodes, liver, and colon by participating in CAMELYON^36^, DigestPath^37^, and PAIP^38^ challenges respectively.

**Figure 1:**
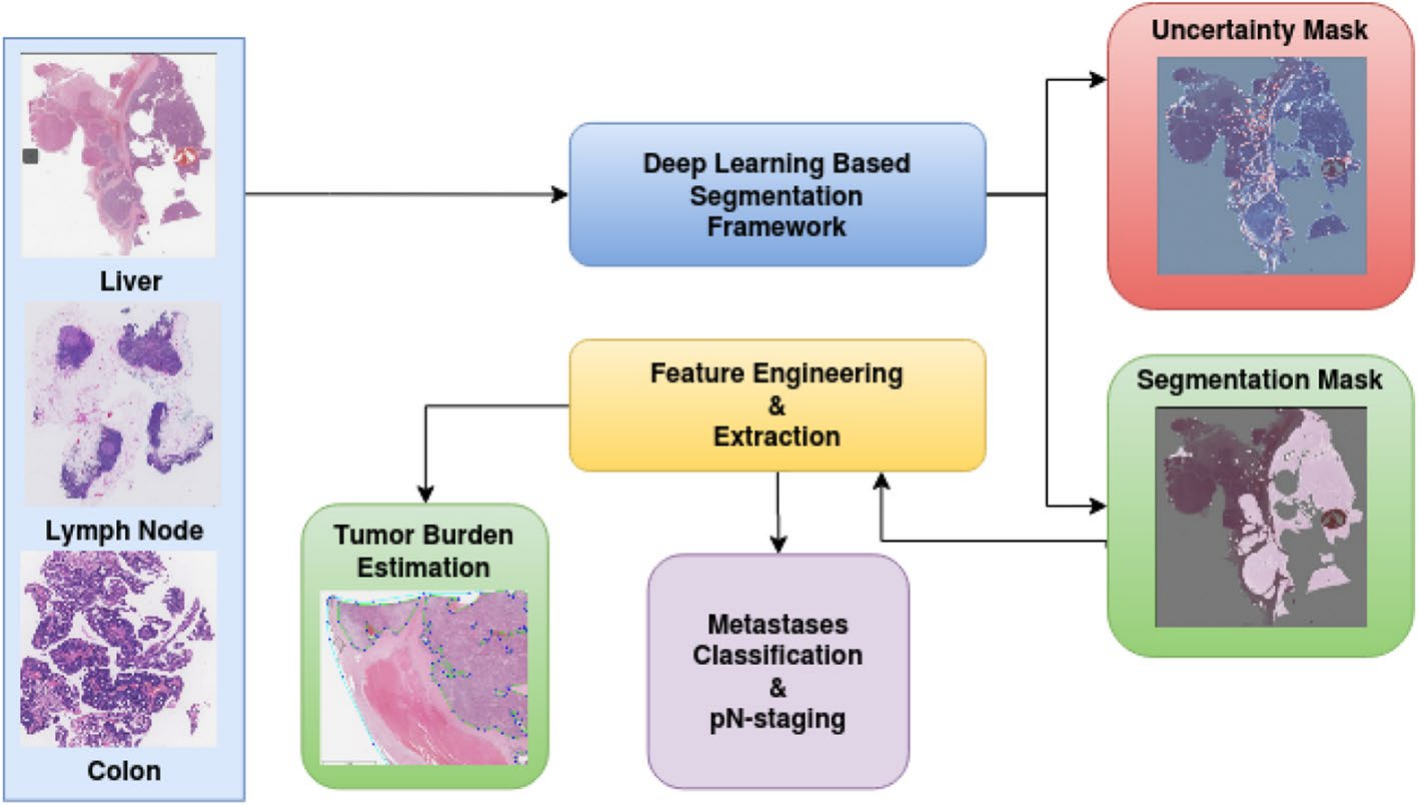
Deep learning based framework for segmentation and analysis of WSI images. Drawn using draw.io (draw.io).

## Materials and methods

This section goes into the details of the proposed framework. Firstly, the ensemble and the network architectures are detailed. Secondly, the strategies used to train the models are explained. Finally, the segmentation inference method is discussed, followed by the methods used to perform secondary histopathology analyses (Viable tumor burden estimation, pn-staging). Figure 1 provides an overview of the proposed deep learning-based segmentation and downstream analyses framework for WSI slide images corresponding to multiple different cancer sites.

### Datasets used for this study

The proposed framework was validated on multiple open-source datasets which included CAMELYON^36^ with 1399 WSI images with an average image size of $100000 \times 100000$ and image resolution of 0.25*microns*/*pixel*, PAIP^38^ with 90 WSI images with an average image size of $50000 \times 50000$ and image resolution of 0.5*microns*/*pixel*, and finally DigestPath^37^ dataset consists of 872 tissue images with image size of $5000 \times 5000$. Table 1 provides an overview of the datasets used in this study.

**Table 1:** Summary of histopathological datasets used in this work. The test images were hidden by the competition organizers and used only for leaderboard evaluation.

Dataset	Train set	Test set	Image size	Microns/pixel
CAMELYON16	270	129	100,000 × 100,000	0.25
CAMELYON17	500	500	100,000 × 100,000	0.25
DigestPath	660	212	5000 × 5000	0.25
PAIP	50	40	50,000 × 50,000	0.5

#### CAMELYON16

The CAMELYON16^25^ dataset comprised of 399 WSI slide images taken from two medical centers in the Netherlands, out of which 159 WSI images were metastases, and the remaining 240 were negative. Pathologists exhaustively annotated all the WSI images with metastases at the pixel level. In the CAMELYON16 challenge, the 399 WSI images were split into training and testing sets, comprising of 160 negative and 110 metastases WSI images for training, 80 negative and 49 metastases WSI images for testing.

**Table 2:** Tumour size criteria for assigning metastasis type.

Category	Size
Isolated tumour cells	Single tumour cells or a cluster of tumour cells not larger than 0.2 mm or less than 200 cells
Micro-metastasis	Larger than 0.2 mm and/or con- taining more than 200 cells, but not larger than 2 mm
Macro-metastasis	Larger than 2 mm

#### CAMELYON17

The CAMELYON17^39^ dataset consisted of 1000 WSI images taken from five medical centers in the Netherlands. In the CAMELYON17 challenge, 500 WSI images were allocated for training, and the remaining 500 WSI images were allocated for testing. The training dataset of CAMELYON17 included 318 negative WSI images and 182 WSI images with metastases. In the CAMELYON17 dataset, slide-level labels of metastases type were provided for all the WSI images, and exhaustive pixel-level annotations were provided for 50 WSI images. The slide-level labels were negative, Isolated tumor cells (ITC), micro-metastases, and macro-metastases. Table 2 provides the size criteria for metastases type. The pN-stage labels were provided for all the 100 patients in the training set and were based on the simplified rules provided in Table 3. Table 4 provides the metastases type distribution in CAMELYON17 training dataset.

**Table 3:** Pathologic lymph node classification (pN-stage) in CAMELYON17 Challenge.

pN-Stage	Slide labels
pN0	No micro-metastases or macro-metastases or ITCs found
pN0(i+)	Only ITCs found
pN1mi	Micro-metastases found, but no macro-metastases found
pN1	Metastases found in 1-3 lymph nodes, of which at least one is a macro-metastasis
pN2	Metastases found in 4-9 lymph nodes, of which at least one is a macro-metastasis

**Table 4:** Metastases type distribution in CAMELYON17 training set.

Metastases (Training set)			
Negative	ITC	Micro	Macro
318	35	64	88

#### PAIP

The PAIP 2019^38^ dataset contains a total of 100 WSI images scanned from liver tissue samples. Each image has an average dimension of 50,000x50,000 pixels. All the images were H&E stained, scanned at 20x magnification, and prepared from a single center (Seoul National University Hospital). The dataset included pixel-level annotation of the viable tumor and whole tumor regions. It also provided the viable tumor burden metric for each image.

Tumor burden is defined as the ratio of the viable tumor region to the whole tumor region. The viable tumor region is defined as the cancerous region. The whole tumor area is defined as the outermost boundary enclosing all the dispersed viable tumor cell nests, tumor necrosis, and tumor capsule . Each tissue sample contains only one whole tumor region. This metric has applications in assessing the response rates of patients to cancer treatment.

Out of the 100 images, 50 images were the publicly available training set, ten images were reserved for validation set that was made publicly available after the challenge was completed, and the rest 40 images were the test set whose ground truth were not publicly available. The test images weren’t used directly in the study. However, the score generated by the PAIP 2019 server by testing our algorithm against the test images was used in the study.

#### DigestPath

The DigestPath dataset consists of tissue sections collected during the examination of colonoscopy pathology to identify early-stage colon tumor cells. There are ten or more tissue sections in a single WSI image for colonoscopy pathology review. The challenge organizers selected one or two tissue sections in a WSI image and provided images of these tissue sections along with their corresponding lesion annotations by pathologists in jpg format. On average, each tissue image was of size 5000 $\times$ 5000 pixels. The training dataset of DigestPath consists of 660 tissue images taken from 324 patients, in which 250 tissue images from 93 patients had lesions, and the remaining 410 tissue images from 231 patients had no lesions. The data was collected from multiple medical centers, especially from several small centers in developing countries. All the tissue sections were H&E stained and scanned at 20 $\times$ magnification. The testing dataset consisted of 212 tissue images from 152 patients. The challenge organizers released only the training set, and the testing set was kept confidential.

### Network architecture

For the task of segmentation of tumor regions from patches of the WSI images, an ensemble of FCN^40^ architectures were used. A typical FCN based segmentation network comprises an encoder network, a decoder network, and a pixel-wise classification layer. An encoder network comprises a series of operations (like convolution and pooling) that transforms the input (image) to a set of low-resolution feature maps. The decoder network comprises of up-sampling or transposed convolution followed by series of convolution operations that transform the low-resolution encoder feature maps to the original input resolution feature maps for pixel-wise classification.

The ensemble consisted of three encoder-decoder-based FCN architectures. Experiments (Tables Experimental 8, 9 in the supplementary note) showed that using an ensemble of three different networks provided superior segmentation performance compared to using the networks individually. During inference, the predicted tumor posterior probability map from all three models was averaged to generate the ensemble model’s final prediction. We carefully selected these three different architectures based on the number of parameters, multi-scale feature extraction, and their performance on PASCAL VOC^41^ open leaderboard. The ensemble comprised of the following FCN architectures:

- U-Net^42^ with DenseNet-121^43^ as the backbone (encoder) pre-trained on ImageNet^44^. The decoder comprised of the bi-linear up-sampling module followed by convolutional layers. Features learned in the down-sampling path of the encoder were concatenated with the features learned in the up-sampling path using skip connection.
- U-Net^42^ with Inception-ResNet-V2^45^ as the backbone (encoder) pre-trained on ImageNet^44^. The Inception-ResNet-V2^45^ (also known as Inception-v4) integrates the features of the Inception architecture^46^ and the ResNet architecture^47^. Multi-scale convnet blocks in inception network helps in reducing number of parameters along with encoding large amount of information.
- DeeplabV3Plus^48^ with Xception^49^ network as the backbone and pre-trained on PASCAL VOC^41^. DeepLabV3^50^ was built to obtain multi-scale context. This was done by using atrous convolutions with different rates. DeeplabV3Plus extends this by having low-level features transported from the encoder to the decoder.

### Training pipeline

**Figure 2:**
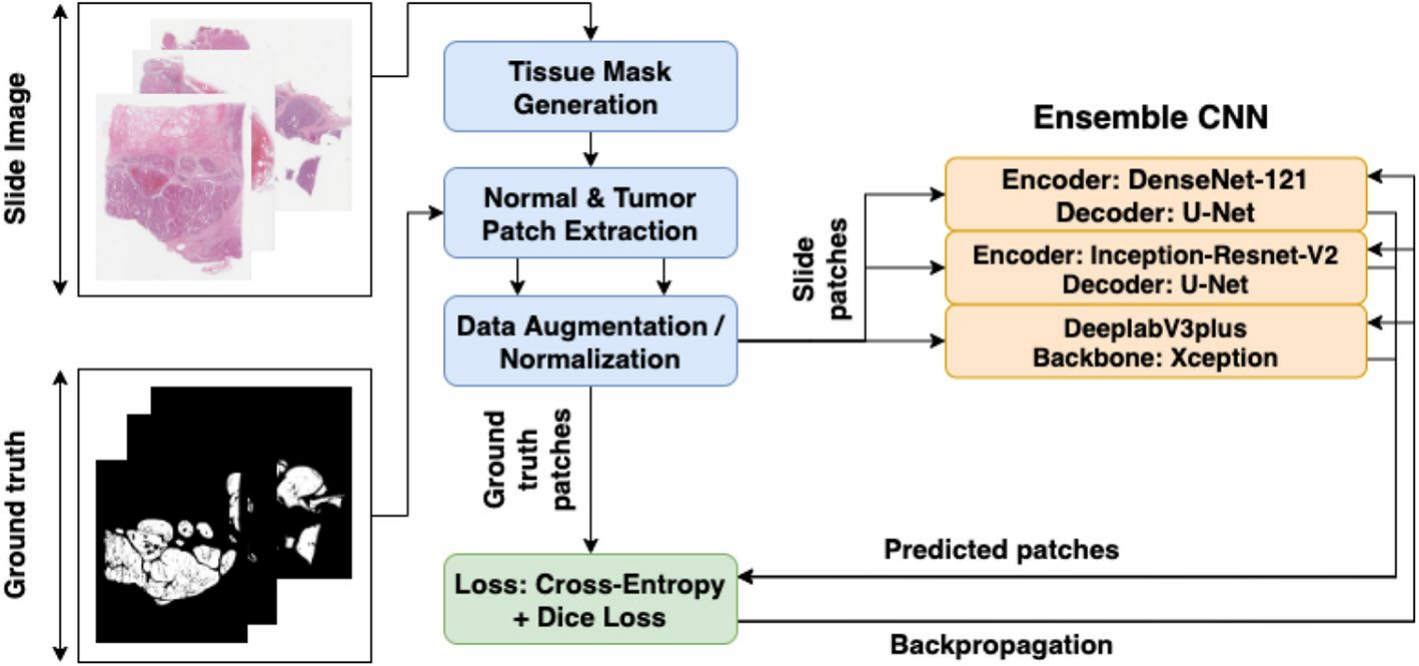
Overview of the tumour segmentation training pipeline. Drawn using draw.io (draw.io).

The training can broadly split into tissue mask generation, patch extraction and training the models patchwise. Figure 2 illustrates the training strategy utilized for training each of the models in the ensemble.

#### Tissue mask generation

In this step, the entire tissue region was segmented from the background glass region of the WSI image. This step aided in preventing unnecessary computations on non-tissue regions of the slide. An approximate tissue region boundary suffices; therefore the processing was done on a low-resolution version of the WSI image to further reduce computational costs. The RGB color space of the low-resolution image was transformed to the HSV (Hue-Saturation-Value) color space and Otsu’s adaptive thresholding^51^ was applied to the saturation component. Post thresholding, binary morphological operations were performed to facilitate proper extraction of patches at the small tissue regions and tissue borders.

**Figure 3:**
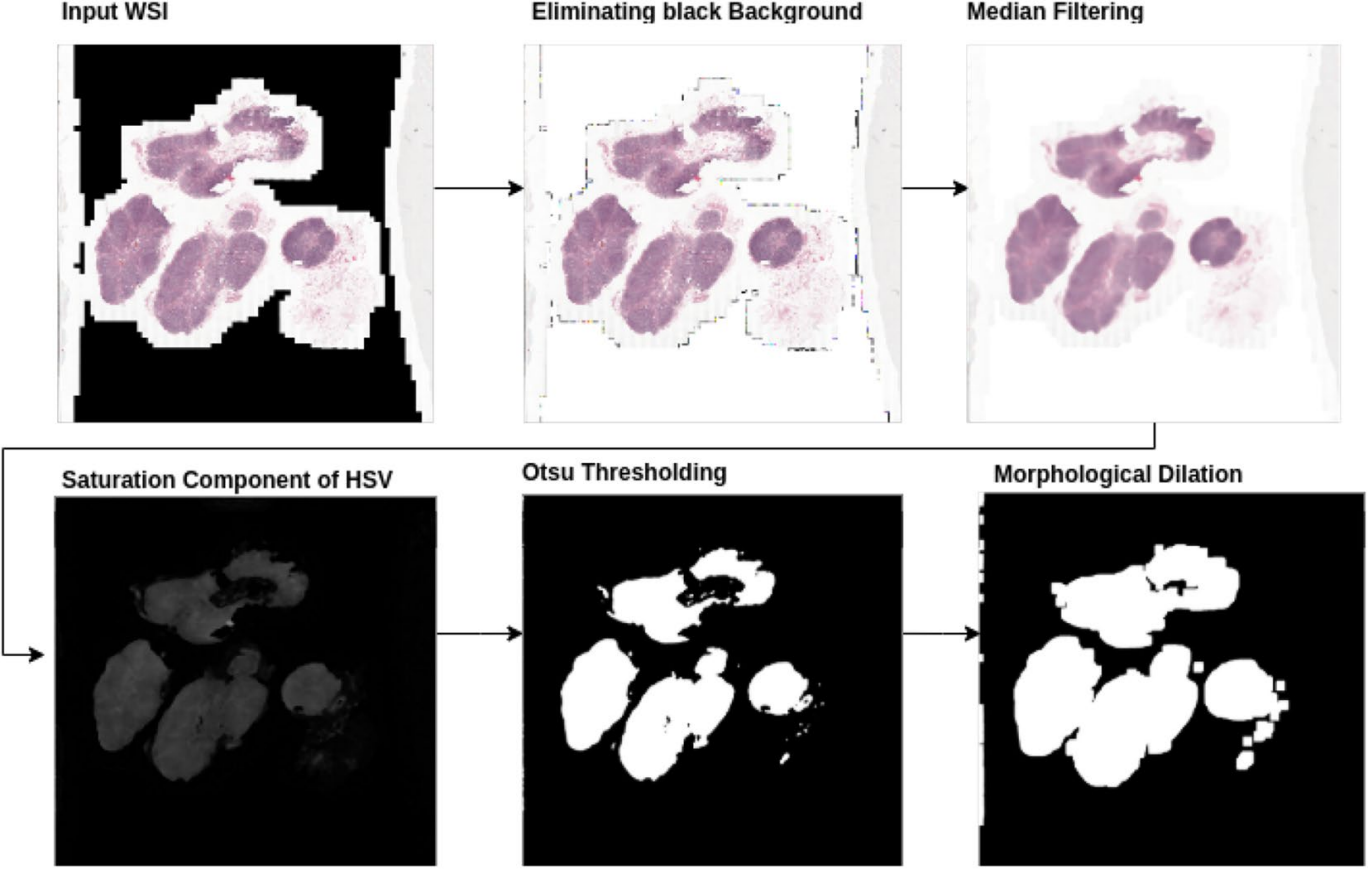
An illustration of the intermediate stages in the process of tissue mask generation from a WSI image in CAMELYON17 dataset. Drawn using draw.io (draw.io).

#### Tissue mask generation specific to CAMELYON dataset

In some of the CAMELYON17 cases, the Otsu’s thresholding failed because of the black regions in the WSI image. Hence, before the application of image thresholding operation, the pre-processing involved replacing black pixel regions in the WSI image background with white pixels and median blurring with a kernel of size 7x7 on the entire image. Median blur aided in the smoothing of the tissue regions and removal of noise at the tissue bordering the glass-slide region while preserving the edges of the tissue. Figure 3 illustrates the pipeline for tissue mask generation with an example.

#### Patch coordinate extraction

Using the tissue mask generated from the previous step, patches of the image were randomly extracted to make the training dataset. An equal number of tumourous and non-tumorous patches were extracted. This was done to prevent class imbalance or manifold shift issues and enforce proper training. A patch was considered tumourous if at least one pixel inside the patch was classified as a tumor. The dimensions of the extracted patches were not fixed; rather, they were a hyperparameter we experimented with. The patches were extracted from the highest resolution of the image.

#### Data augmentation

To increase the number of data points and to better generalize the models across various staining and acquisition protocols, data augmentation schemes were proposed. Augmentations like “horizontal or vertical flip,” “90-degree rotations”, and “Gaussian blurring” along with color augmentation were performed. Colour augmentation included random changes to brightness, contrast, hue, and saturation with a maximum delta of 64.0/255, 0.75, 0.25, 0.04, respectively.

Additionally, in order to introduce some diversity between patches extracted from the images at different epochs, random coordinate perturbation was introduced. This technique involved randomly offsetting the center of the patch within a specified radius (128 pixels) prior to the extraction from the WSI image. Post augmentation, the images were normalized.

#### Loss function

Tumour regions were represented by a minuscule proportion of pixels in WSI images, thereby leading to class imbalance. This issue was circumvented by training the network to minimize a hybrid loss function. The hybrid loss function is comprised of cross-entropy loss and a loss function based on the Dice overlap coefficient. The Dice coefficient is an overlap metric used for assessing the quality of segmentation maps. The effect of hybrid loss was extensively studied in^52^ showing an overall improvement in segmentation performance by combining cross-entropy and dice loss. The dice loss is a differentiable function that approximates Dice-coefficient and is defined using the predicted posterior probability map and ground truth binary image as defined in (1). The cross-entropy loss is defined in (2). In the equations, $p_i$ and $g_i$ represent pairs of corresponding pixel values of predicted posterior probability and ground truth. *N* represents the total number of pixels. *DL* refers to dice loss and *CL* refers to cross-entropy loss. $DL_{FG}$ and $DL_{BG}$ represent the foreground pixels that correspond to the tumor regions and the background pixels that corresponded to non-tumor regions, respectively.

1 $$\begin{aligned} DL= & {} 1 - \frac{2\sum _i ^N p_i g_i}{ \sum _i^N p_i^2+ \sum _i^N g_i^2} \end{aligned}$$

2 $$\begin{aligned} CL= & {} \sum _i ^N \left( g_ilog(p_i) + (1 - g_i)log(1-p_i)\right) \end{aligned}$$

3 $$\begin{aligned} Loss= & {} \alpha *CL + \beta *DL_{BG} + \gamma *DL_{FG} \end{aligned}$$

The proposed loss was defined as a linear combination of the two-loss components as defined in (3). The neural networks were trained by minimizing the proposed loss function using ADAM optimizer^53^. The $\alpha , \beta , \gamma$ were assigned such that the cross-entropy loss and the dice loss are given equal weightage ($\alpha = 0.5, \beta = 0.25$ and $\gamma = 0.25$).

#### Training

All three models were trained independently, with different cross-validation folds of the data. The FCN architectures in the ensemble whose encoders were based on DenseNet-121 and Inception-ResNet-V2 made use of transfer learning by using ImageNet^44^ pre-trained weights for their respective encoders. In the case of DeeplabV3Plus, the model weights were pre-trained on PascalVOC^41^. For the network architectures with encoders based on DenseNet-121 and Inception-ResNet-V2, the encoder weights of the models were frozen for the first two epochs, and only the decoder weights were made trainable. For the remaining epochs, both the encoder and decoder parts were trained. The learning rate was decayed every few epochs in a deterministic manner to allow for the model to gradually converge. The training was stopped when the validation loss between epochs started increasing.

### Inference pipeline

**Figure 4:**
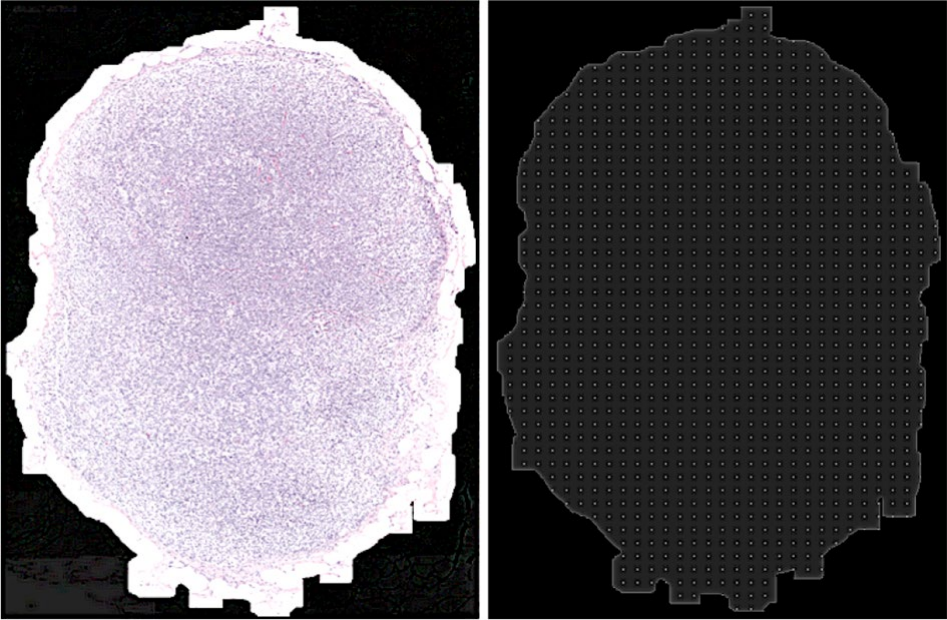
(Left to Right) An illustration of the tissue mask overlayed on a small region of the WSI image at low resolution (level-4), here the white region corresponds to the tissue mask; An illustration of the generated uniform patch coordinate sampling grid, here the points on the image act as centers from which high-resolution image patches were extracted from the WSI image.

The pre-processing step in the inference pipeline included segmentation of tissue region from the WSI image (refer “Training pipeline” section). In order to facilitate extraction of patches from the WSI image within the tissue mask region, a uniform patch-coordinate sampling grid was generated at a lower resolution, as shown in Fig. 4. Each point in the patch sampling grid was re-scaled by a factor to map to the coordinate space corresponding to the WSI image at its highest resolution. With these scaled coordinate points as the center, fixed-size high-resolution image patches were extracted from the WSI image for feeding the trained segmentation model as an input.

The sampling stride was defined as the spacing between consecutive points in the patch sampling grid. The patch size and the sampling stride controlled the overlap between consecutive extracted patches from the WSI image. The main drawback of the patch-based segmentation method for WSI images was that the smaller patch sizes could not capture the wider context of the neighborhood region. Moreover, the stitching of the segmented patches introduced boundary artifacts (*blockish* appearance) in the tumor probability heatmaps. The generated heatmaps were smooth when the inference was done on overlapping patches with larger patch-size and averaging the prediction probabilities at the overlapping regions. The experimental observation suggested that a 50% overlap between consecutive neighboring patches is the optimal balance between accuracy and computational efficiency. Also, during inference, increasing the patch size by a factor of 4 (1024x1024) when compared to the patch size used during training (256x256) improved the quality of generated heatmaps.

### pN-staging pipeline for CAMELYON17 dataset

**Figure 5:**
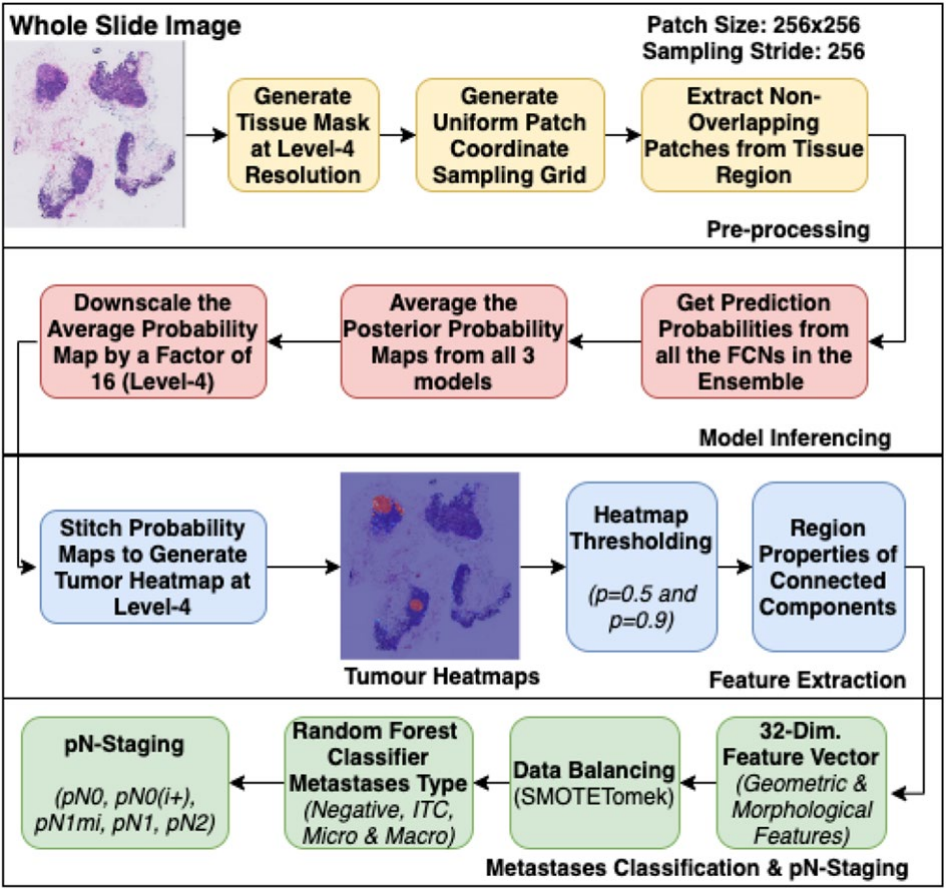
Overview of the steps involved in the pN-staging pipeline developed for CAMELYON17 dataset. Drawn using draw.io (draw.io).

**Table 5:** List of features extracted for the purpose of predicting lymph node metastases type. Features were extracted after thresholding tumour probability heatmaps. For feature numbers 5, 6, 7, 8 and 9 the following statistics were computed- maximum, mean, variance, skewness, and kurtosis.

No.	Feature description	Threshold (*p*)
1	Largest tumour region’s major axis length	$p=0.9$ & $p=0.5$
2	Largest tumour region’s area	$p=0.5$
3	Ratio of tumour region to tissue region	$p=0.9$
4	Count of non-zero pixels	$p=0.9$
5	Tumour regions area	$p=0.9$
6	Tumour regions perimeter	$p=0.9$
7	Tumour regions eccentricity	$p=0.9$
8	Tumour regions extent	$p=0.9$
9	Tumour regions solidity	$p=0.9$
10	Mean of all region’s mean confidence probability	$p=0.9$
11	Number of connected regions	$p=0.9$

Figure 5 illustrates the complete pipeline developed for pN-staging of CAMELYON17 dataset. The pipeline comprises four blocks as described below:

- Pre-processing: The tissue regions in the WSI images were detected for patch extraction.
- Heatmap generation: The extracted patches from the WSI images were passed through the inference pipeline to generate the down-scaled version of the tumor probability heatmaps.
- Feature extraction: The heatmaps were binarized by thresholding at 0.5 and 0.9 probabilities, and at each of these thresholds, the connected components were extracted, and region properties were measured using scikit-image^54^ library. Thirty-two geometric and morphological features from the probable metastases regions were computed (Table 5).
- Data balancing: In order to handle the class imbalance problem, one of the techniques proposed in the literature is oversampling by synthetically generating minority class samples using SMOTE algorithm^55^. However, this method can introduce noisy samples when the interpolated new samples lie between marginal outliers and inliers. This problem is usually addressed by removing noisy samples by using under-sampling techniques like Tomek’s link^56^ or nearest-neighbors. SMOTETomek^57^ algorithm was employed for balancing the training data. SMOTETomek algorithm is a combination of SMOTE and Tomek’s link performed consecutively.
- Classification: The pN-stage was assigned to the patient based on all the available lymph-node WSI images, taking into account their individual metastases type (Table 2). For predicting the metastases type, an ensemble of Random Forest classifiers^58^ was trained using the extracted features.

### Tumour burden estimation for PAIP dataset

The tumor burden computation requires the segmentation of the viable tumor and whole tumor regions in the WSI image of the liver cancer tissue. The viable tumor region was segmented using the proposed deep learning-based segmentation network. However, it was observed that training the same segmentation network for the whole tumor region gave sub-optimal results. Hence, a heuristic method was adopted to approximate the whole tumor region from the viable tumor region.

The tumor burden estimation algorithm consisted of the following steps:

- Segment the viable tumor region via the proposed algorithm in “Inference pipeline” section
- Apply morphological operations on the prediction to remove false positives and fill the small holes
- Find the smallest convex hull containing the entire viable tumor region
- Estimate the tissue mask, as discussed in “Training pipeline” section
- The whole tumor region is approximated to be the intersection of the convex hull and tissue mask region
- The tumor burden is calculated by taking the ratio between the area of the viable and whole tumor regions

### Uncertainty analysis

Uncertainty estimation is essential in assessing unclear diagnostic cases predicted by deep learning models. It helps pathologists to concentrate more on the uncertain regions for their analysis. Begoli et al.^59^ argues the need for uncertainty analysis in machine-assisted medical decision-making system. There exist two main sources of uncertainty, namely (i) Aleatoric uncertainty and (ii) Epistemic uncertainty. Aleatoric uncertainty is uncertainty due to the data generation process itself. In contrast, the uncertainty induced due to the model parameters, which is the result of not estimating ideal model architectures or weights to fit the given data, is known as epistemic uncertainty^60^. Epistemic uncertainty can be approximated by using test time Bayesian dropouts^61^, which estimates uncertainty by Montecarlo simulations with Bayesian dropout.

In the proposed pipeline, aleatoric uncertainty for each model was estimated using test time augmentations, as introduced in^62^ (4).

4 $$\begin{aligned} var_{al}(x, \Phi _i) \approx {\mathbf {E}}_{t \sim TTA}[(\Phi _i(x|w,t) - {\mathbf {E}}_{t \sim TTA}[\Phi _i(x|w,t)])^2] \end{aligned}$$

where $\Phi _i(x|w)$ is the output of the neural network with weights *w* for input *x* and *TTA* denotes the set of possible test time data augmentations allowed. The proposed methodology for aleatoric uncertainty included the following augmentations- $TTA \in \{rotation, vertical flip, horizontal flip\}$.

For epistemic uncertainty, the diversity of model architectures were used to calculate uncertainty (5).

5 $$\begin{aligned} var_{ep}(p(y|x,w)) \approx {\mathbf {E}}_{\phi \sim \{\Phi _i\}}[(\phi (x|w) - {\mathbf {E}}_{\phi \sim \{\Phi _i\}}[\phi (x | w)])^2] \end{aligned}$$

where the likelihood distribution *p*(*y*|*x*, *w*) is a probabilistic model which generates outputs (*y*) for given inputs (*x*) for some parameter setting (*w*) and $\Phi _i$ indicates the trained model.

## Challenge results

### Performance evaluation on CAMELYON17 challenge

On the CAMELYON17 testing dataset (n=500) the ensemble strategy was employed by combining the predictions from all the four trained Random Forest classifiers. The ensembling was based on the majority voting principle, and in case of a tie, the higher metastases category was selected. The ensemble model is referred to as RF-Ensemble. Table 6 compares the results of the proposed ensemble approach with other published approaches on the CAMELYON17 testing dataset (n=500). The proposed ensemble strategy gave Cohen’s kappa score of 0.9090.

**Table 6:** Comparison of the proposed with other published approaches for automated pN-Staging in CAMELYON17 challenge. The score reported in the table is from the open public leader board of the CAMELYON17 challenge. The proposed approach (RF-Ensemble) stood rank-3 on the leaderboard (Accessed on 31-Dec-2019). The table additionally provides the performance of individual Random Forest classifiers in the ensemble and RF-Ensemble classifier.

Method	Cohen Kappa Score	Rank
Lee et al.^63^	0.9570	1
Pinchaud^64^	0.9386	2
**Proposed (RF-Ensemble)**	0.9090	3
Proposed (RF-PI)	0.8971	12
Proposed (RF-PB)	0.9027	9
Proposed (RF-CI)	0.8889	18
Proposed (RF-CB)	0.9057	6

### Performance evaluation on DigestPath 2019 challenge

Table 7 compares the results of the proposed with other approaches on DigestPath-2019 testing dataset (n=212). The proposed approach obtained a Dice score of 0.78 on the test set. Though the proposed method is ranked fourth, it can be observed that the results of all the top three methods lie inside the estimated confidence bounds of $\pm 0.014$ as described in supplementary section 1.6.

**Table 7:** Top four entries in DigestPath-2019 challenge.

Teams	Dice
kuanguang	0.807
zju_realdoctor	0.792
TIA_Lab	0.787
**Proposed**	0.782

### Performance evaluation on PAIP 2019 challenge

Table 8 compares the results of the proposed with other approaches on PAIP-2019 testing dataset (n=40). The challenge comprised of two tasks, described as follows-

- Task 1: Liver cancer segmentation performance was evaluated using the average Jaccard index.
- Task 2: Viable tumor burden estimation was evaluated as the average of products of absolute accuracy and corresponding Task 1 score (Jaccard index) for each of the cases in the test set.

For Task 1, all the participants utilized deep learning-based methods for the segmentation of viable tumors, albeit with different CNN architectures. For Task 2, all the participants used deep learning-based methods for the segmentation of the whole tumor. The proposed convex hull-based approximation method showed comparable performance with deep learning-based methods.

**Table 8:** Top five entries of PAIP 2019. Task 1 corresponds to Viable tumour segmentation and Task 2 corresponds to Viable tumour burden estimation. Note: FNLCR: Frederick National Laboratory for Cancer Research.

Team	Task 1	Task 2
FNLCR	0.789	0.752
Sichuan University	0.777	NA
**Proposed**	0.750	0.6337
Alibaba	0.672	0.6199
Sejong University	0.665	0.6330

## Discussion and conclusions

An automated end-to-end deep learning-based framework for segmentation and downstream analysis of WSI images was developed. The proposed framework showed state-of-the-art results on three publicly available histopathology image analysis challenges, namely, CAMELYON, PAIP 2019, and DigestPath 2019. The problem of segmentation of gigapixel WSI images was approached using the divide-and-conquer strategy by dividing the large image into computationally feasible patch sizes, running segmentation algorithms on the extracted patches, and stitching the individual outputs together to generate the segmentation of the entire WSI image. The patches were segmented using an ensemble of FCNs, which are encoder-decoder-based architectures employed for generating dense pixel-level classification. The encoders in the proposed FCNs were some of the state-of-the-art CNNs used for natural image analysis tasks, and the decoders were a learnable upsampling module to generate dense predictions. The proposed segmentation framework was an ensemble comprising of multiple FCN architectures, each independently trained on different subsets of the training data. The ensemble generated the tumor probability map by averaging the posterior probability maps of all the FCNs. The ensemble approach showed superior segmentation performance when compared to its individual constituting FCNs. The patch-based segmentation methods for large-sized images suffer from loss of neighboring context information at patch borders. This issue was addressed during inference by proposing- (i) to use patch size larger than that used during training and (ii) to overlap patches and average the posterior probabilities of the overlapping regions while stitching the output together. In addition to the generation of tumor probability heatmaps, a methodology for generating uncertainty maps based on model and data variability was also incorporated into the framework. These uncertainty maps would assist in better interpretation by pathologists and fine-tuning the model with uncertain regions.

Further research can be done in the design of efficient and multi-resolution FCN architectures for capturing multi-resolution information from WSI images^65^. The proposed experimental analysis on transfer learning showed that pre-training models with different histopathology datasets could act as good starting points for training models where pathology datasets are limited. Post-processing techniques could be one of the directions to improve the predicted WSI image’s tumor segmentation; techniques such as patch-based conditional random fields^66,67^ could be employed to refine the predicted segmentation masks rather than employing hardcoded threshold values. In the current study, the presence of artifacts in WSI images makes it difficult for tissue region sampling, which further results in sub-optimal segmentation results. The addition of a pre-processing stage for filtering these artefacts, or the addition to the training set of a significant number of images that include these elements, could lead to an improvement in the robustness of the framework. Moreover, the majority of the images used in this study were stained using H&E stain. Increasing the heterogeneity of the training samples with other possible stains could increase the generalizability of the framework.

The segmentation of WSI images is usually the first step which precedes other specific analyses such as metastases classification and estimation of tumor burden. In this regard, an automated pipeline for lymph node metastases classification and pN-staging was developed. For the task of lymph node metastases classification, an ensemble of multiple Random Forest classifiers was proposed, and each classifier was trained on different subsets of the training data. The training data was prepared by extracting features based on the pathologist’s viewpoint from the tumor probability maps. Additionally, incorporating synthetically generated training samples into the training data demonstrated its efficacy in addressing class imbalanced datasets for such classification tasks.

The proposed method for viable tumor burden estimation from WSI images of liver cancer utilized an empirical method for estimating the whole tumor region from the predicted viable tumor region. The whole tumor region was proposed to approximate a convex hull around the viable tumor region. This approximation performed on par with other deep learning-based segmentation approaches and was also computationally inexpensive. The proposed method could be refined further by incorporating learning-based methods into the empirical method. For example, the convex hull output could be used as an initial point for active contours-based models^68^ for correcting whole tumor region segmentation.

## Supplementary information

Supplementary Information.
