# Supplementary material for: A generalized deep learning framework for whole-slide image segmentation and analysis
Source: Sci Rep. 2021 Jun 2;11:11579. doi: 10.1038/s41598-021-90444-8 (PMC8172839; doi:10.1038/s41598-021-90444-8)
Supplement: Supplementary file 1 — Supplementary Information. [file 41598_2021_90444_MOESM1_ESM.pdf]

A Generalized Deep Learning Framework for Whole-Slide Image Segmentation and Analysis

Mahendra Khened[1,+], Avinash Kori[1,+], Haran Rajkumar[1,+], Ganapathy Krishnamurthi[1,*], and Balaji Srinivasan[2,]

[1]Department of Engineering Design, Indian Institute of Technology Madras, Chennai, 600036, India
[2]Department of Mechanical Engineering, Indian Institute of Technology Madras, Chennai, 600036, India
[+]these authors contributed equally to this work
[*]Correspondence to: gankrish@iitm.ac.in

ABSTRACT

In this supplementary note, we discuss the experimental analysis of the methods described in the main paper. We analyze the methods with the 3 datasets, PAIP2019, CAMELYON, DIGESTPATH2019.

1 Experimental analysis

In this section, the effectiveness of the proposed methodologies for segmentation and classification models are experimentally analyzed. The neural networks were implemented using TensorFlow-Keras ([1]) deep learning framework. The experiments were run on multiple desktop computers with NVIDIA Titan-V GPU with 12 GB RAM, Intel Core i7-4930K 6-core CPUs @ 3.40GHz, and 48GB RAM. The architecture of 3 models used are detailed in Figures 1, 2, and 3.

1.1 Lesion detection analysis on CAMELYON16 dataset

In this section, some of the techniques specific to CAMELYON dataset pre-processing are detailed, and discussion on the performance of various FCN architectures and ensemble configurations for lesion detection on the CAMELYON16 test dataset (n=139) are provided.

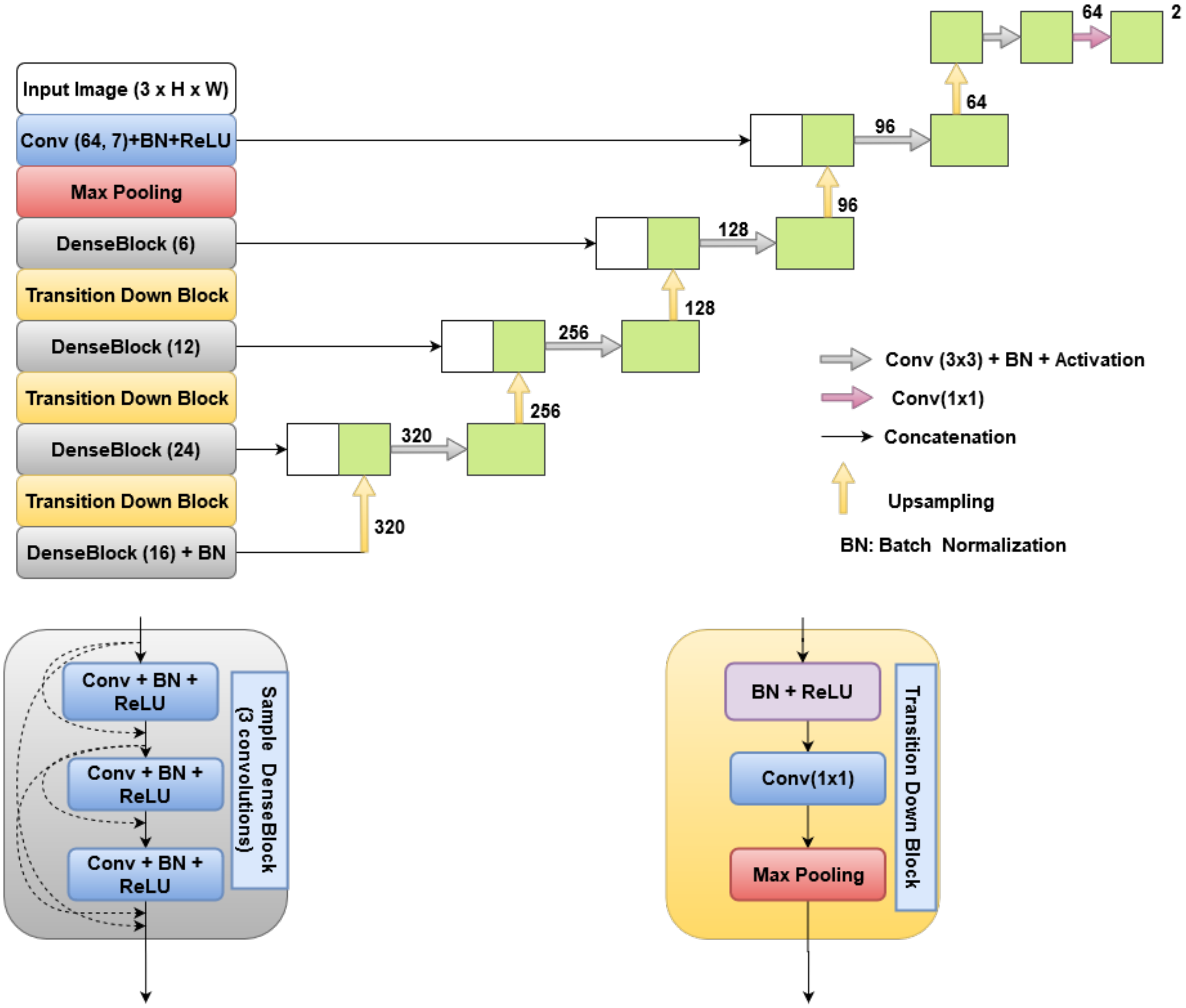

Figure 1. DenseNet-121 based FCN. Drawn using draw.io (draw.io).

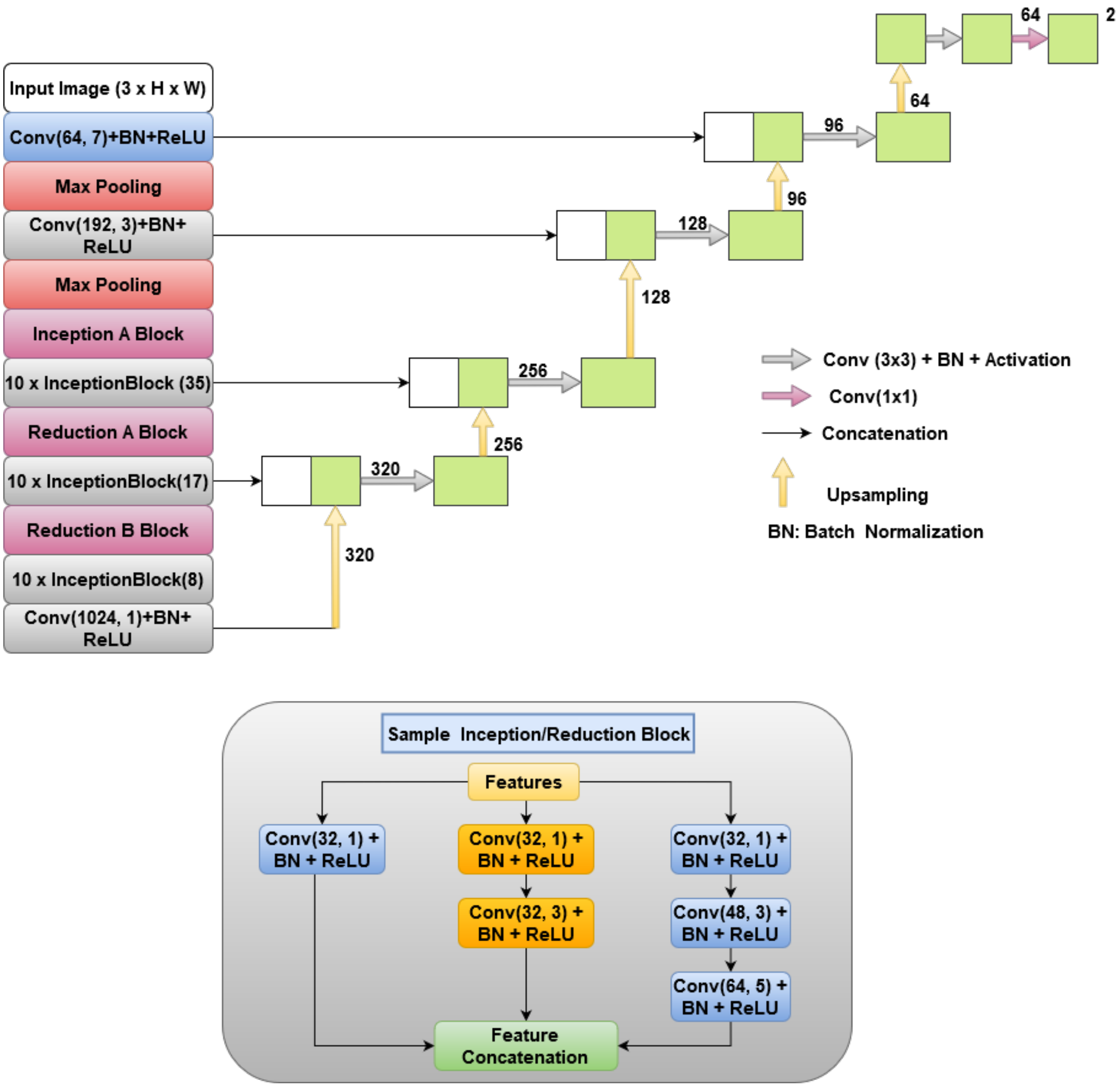

Figure 2. Inception-ResNet-V2 based FCN. Drawn using draw.io (draw.io).

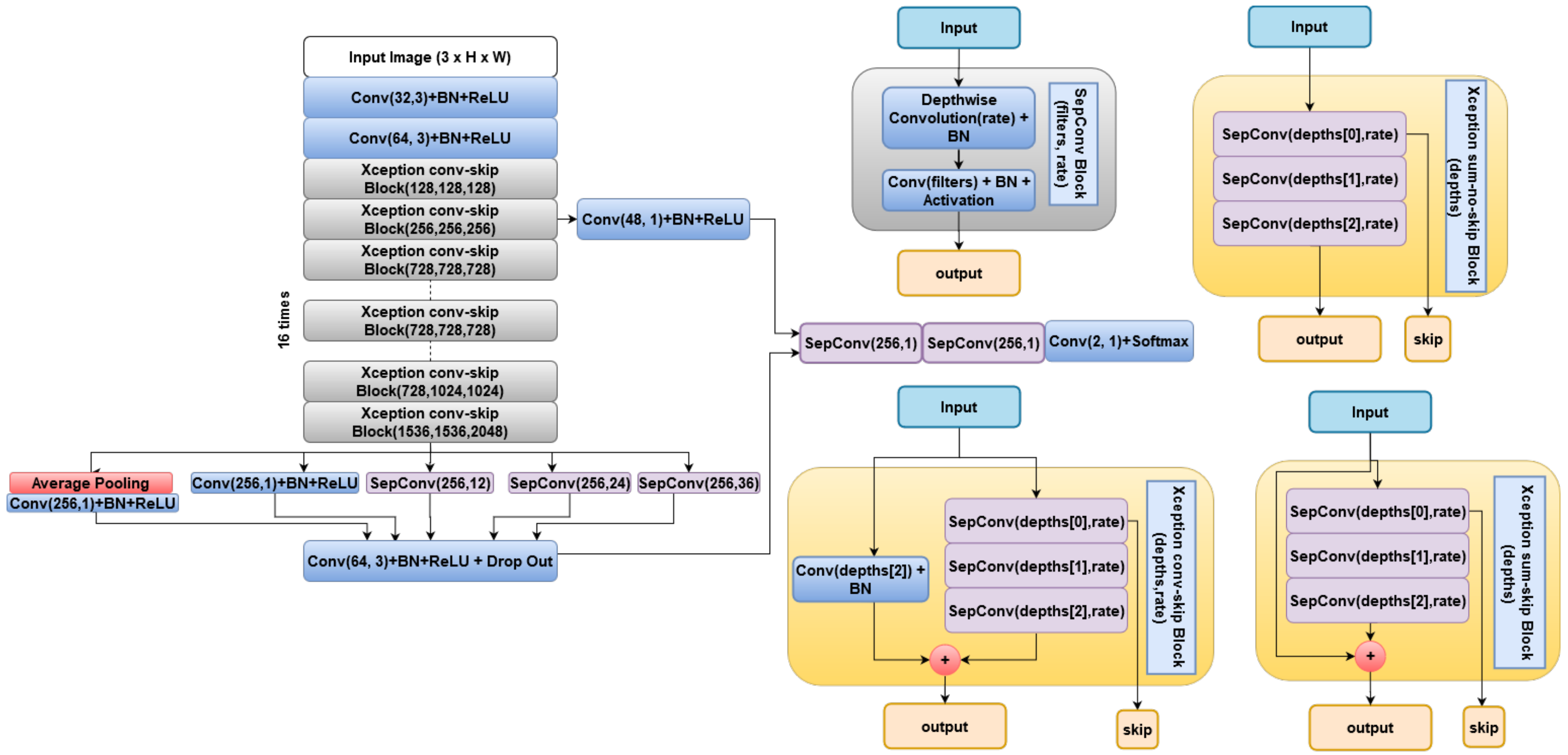

Figure 3. DeepLabV3Plus architecture. Drawn using draw.io (draw.io).

1.1.1 FROC evaluation score

One of the metrics used in the CAMELYON16 challenge for lesion-based evaluation is the free-response receiver operating characteristic (FROC) curve. The FROC curve is defined as the plot of sensitivity versus the average number of false positives per image. The CAMELYON16 challenge testing dataset was used for evaluating the performance of the proposed algorithms for lesion detection/localization. The detection/localization performance was summarized using Free Response Operating Characteristic (FROC) curves. This was similar to ROC analysis, except that the false positive rate on the x-axis is replaced by the average number of false positives per WSI image. The following guidelines were followed for lesion detection in the CAMELYON16 challenge.

- If the position of the detected region was inside the annotated ground truth lesion it was considered a true positive
- If a single ground-truth region had several findings, they were counted as a single true positive finding, and none of them were counted as false positives
- All detections which were not within a reasonable distance of the annotations of ground truth were counted as false positives
- The final FROC score was defined as the average sensitivity at six predefined false positives: 1/4, 1/2, 1, 2, 4, and 8 FPs per WSI image

1.1.2 Dataset preparation specific to CAMELYON dataset

For training the ensemble segmentation model for lesion segmentation, training sets of both CAMELYON16 and CAMELYON17 datasets which had pixel-level annotations for the WSI images were used. As noted by the challenge organizers, some of the WSI images were not exhaustively annotated in the CAMELYON16 training set; such slides were excluded in training set preparation. So, in total, 628 WSI images for training were utilized (250 WSI images from CAMELYON16 and 378 from CAMELYON17). A three-fold stratified cross-validation split of the training set was done to maximize the utilization of the limited number of WSI images. The stratification ensured that the ratio of negative to metastases was maintained in all three folds. From 628 WSI images, 5,71,029 coordinates whose patches corresponded to regions from the tumor and non-tumor tissue regions were randomly sampled. A patch extracted from a WSI image was labeled as a tumor patch if it had non-zero pixels labeled as tumor pixels in the pathologist's manual annotation. Further, these extracted patch coordinates were distributed into their respective cross-validation folds. Table 1 shows the distribution of the split in each of the folds.

Table 1. Count of the tumor and non-tumor patches in each of the three cross-validation folds.

Patch label	No. of patch coordinates		
	**Fold-0**	**Fold-1**	**Fold-2**
Training Non-Tumour	1,87,034	1,96,094	1,90,424
Training Tumour	1,84,467	1,94,709	1,87,440
Validation Non-Tumour	99,742	90,682	96,352
Validation Tumour	99,786	89,544	96,813

1.1.3 Training and inference configuration of ensemble FCN models

The following two ensemble configurations were proposed:

- Ensemble-A: Comprised of the three different FCN architectures, as described in the methods section of the paper. The inference pipeline made use of a patch size of 256 and extracted non-overlapping patches.
- Ensemble-B: Comprised of three replicated versions of a single FCN architecture. The inference pipeline made use of a patch size of 1024 with a 50% overlap between neighboring patches, as illustrated in Figure 4.

In both, the ensemble configurations, each model in the ensemble was trained with one of the 3-fold cross-validation splits. All the models made use of pre-trained weights with the fine-tuning procedure, as described in the methods section (Section 3.3). The models were trained for ten epochs with a batch size of 32.

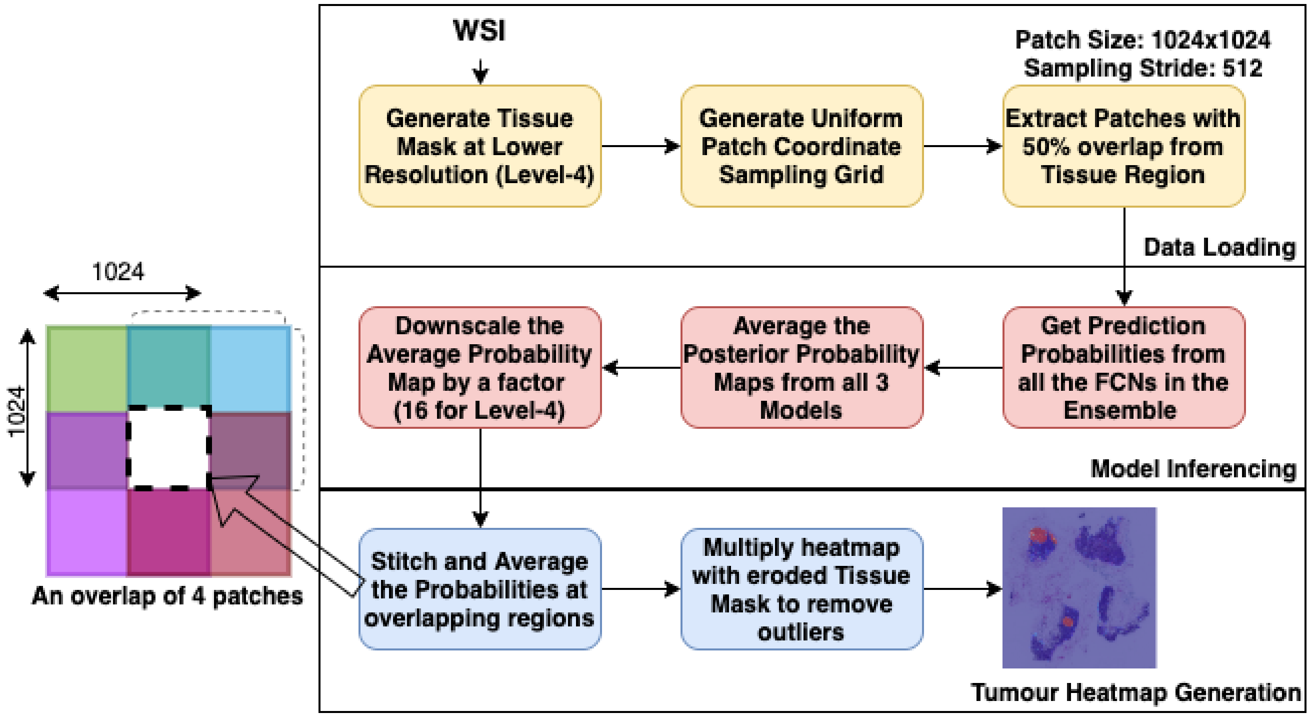

Figure 4. The figure illustrates the overlap-stitch inference pipeline used in Ensemble-B configuration. Drawn using draw.io (draw.io).

1.1.4 Lesion detection performance of Ensemble-A

Experimental results suggested that DenseNet-121 architecture had higher sensitivity and reduced false positives when compared to other FCNs in the ensemble configuration. It was also observed that Ensemble-A showed a significant difference in the FROC score compared to its constituents. The reason for this significant boost in the performance of Ensemble-A could be attributed to the effect of averaging the heatmaps from multiple FCN models, thereby lowering the probabilities of uncertain or less confident regions and hence eliminating the false positives. Figure 6 illustrates the heatmaps generated by Ensemble-A and its constituent FCN models on a CAMELYON16 test case (refer Fig. 5).

Table 2. FROC scores achieved on CAMELYON16 test set (n=139) by FCN models in Ensemble-A configuration. Note the abbreviations: IRF - Inception-ResNet-V2 FCN, DF- DenseNet-121 FCN, DL- DeepLabV3Plus, FP- false positives.

Avg. FPs /Slide	Sensitivity			
	IRF(Fold-0)	DF(Fold-1)	DL(Fold-2)	Ensemble-A
0.25	0.5	0.59	0.03	0.77
0.5	0.59	0.65	0.06	0.8
1	0.69	0.72	0.2	0.83
2	0.77	0.79	0.48	0.84
4	0.8	0.83	0.64	0.86
8	0.82	0.85	0.77	0.86
**FROC Score**	0.69	0.74	0.36	**0.83**

1.1.5 Lesion detection performance of Ensemble-B

Since there was a marginal difference in FROC scores between various ensemble configurations (Table 4); hence in the interest of minimizing the computation time, Ensemble-A configuration was preferred with a patch-size of 256x256 and sampling stride set to 256 (non-overlapping patch acquisition) for running inference on CAMELYON17 testing dataset (n=500).

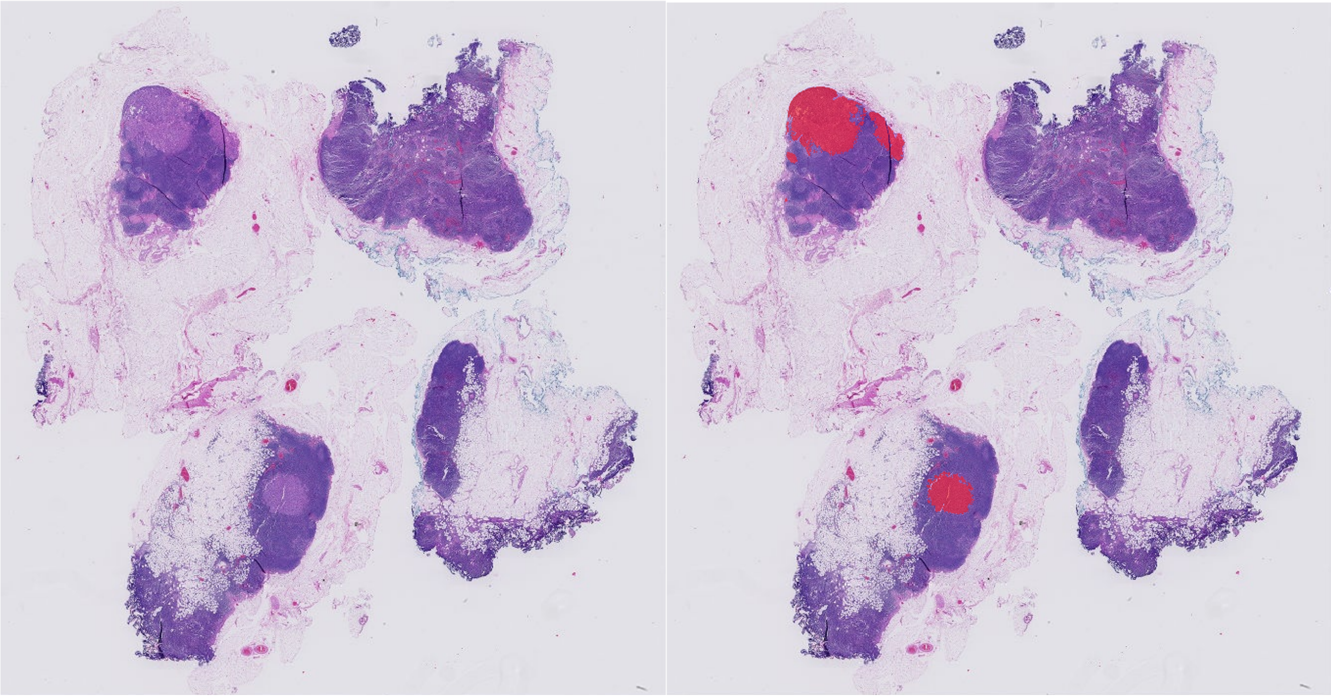

Figure 5. Left image describes a WSI image from CAMELYON16 test set, and the right image shows the tumour ground truth overlayed on the WSI image. Generated using Matplotlib 3.1.1 (https://matplotlib.org/).

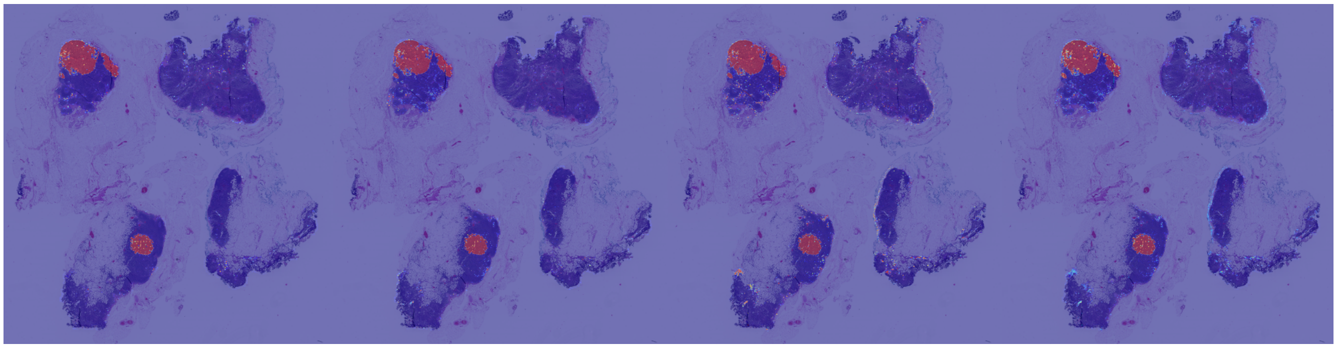

Figure 6. The figure shows the heatmaps overlayed on the WSI image by FCN models in Ensemble-A configuration. (Left to Right): DenseNet-121 FCN, Inception-ResNet-V2 FCN, DeepLabV3plus, Ensemble, (Patch Size: 256x256, Sampling Stride: 256 pixels). Generated using Matplotlib 3.1.1 (https://matplotlib.org/).

Table 3. FROC scores achieved on CAMELYON16 test set (n=139) by FCN models in Ensemble-B configuration (patch size-1024 and sampling stride- 512). Note the abbreviations: DF-DenseNet-121 FCN, FP-false positives, F-Fold.

**Avg. FPs /Slide**	**Sensitivity**			
	**DF (F-0)**	**DF (F-1)**	**DF (F-2)**	**Ensemble-B**
0.25	0.56	0.56	0.61	0.77
0.5	0.65	0.63	0.69	0.84
1	0.71	0.70	0.76	0.85
2	0.77	0.75	0.81	0.88
4	0.82	0.80	0.84	0.88
8	0.86	0.86	0.88	0.89
**FROC Score**	0.73	0.72	0.77	**0.85**

Table 4. The table shows the FROC scores on CAMELYON16 test set (n=139) for various configurations of model, patch-size, and sampling-stride.

**Model**	**Patch size**	**Sampling stride**	**FROC score**
Ensemble-A	256	256	0.83
Ensemble-B	256	256	0.84
Ensemble-B	1024	1024	0.86
Ensemble-B	1024	512	0.85

1.2 Lymph-node metastases type classification analysis on CAMELYON17 dataset

In this section, the experimental analysis of the classification model for lymph node metastases types is presented.

1.2.1 Cohen's kappa evaluation score

Cohen's kappa[2] is a statistic that measures the inter-rater reliability for categorical variables. In the CAMELYON17 challenge for evaluating pN-staging of the patients, the metric used was Cohen's kappa with five classes and quadratic weights. The kappa metric ranges from -1 to +1, where 1 represented perfect agreement with the raters, and 0 represented the amount of agreement that can be expected by random chance and, a negative value represented lower than chance agreement.

1.2.2 Dataset preparation

The CAMELYON17 training dataset had 100 patients, and each patient had five WSI images with their corresponding metastases labels (total 500 slide images). The training dataset comprising 100 patients was split into 43 patients as a train set and the

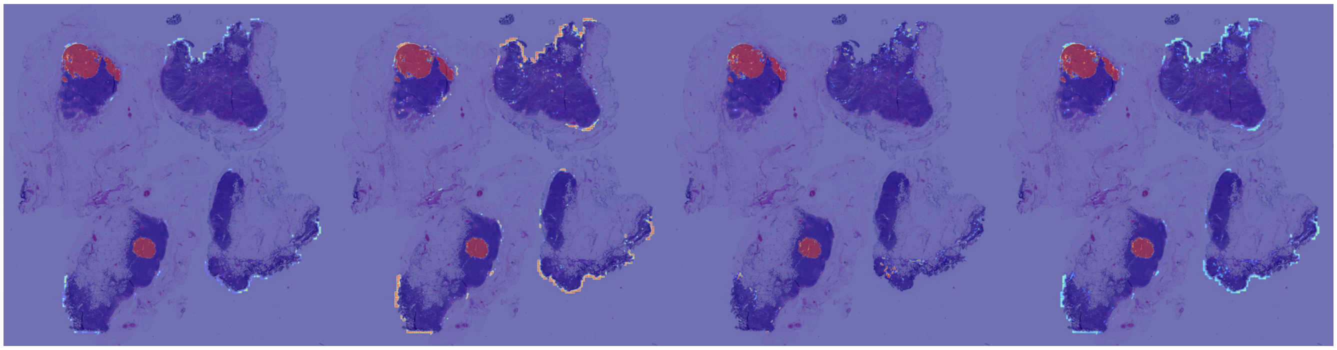

Figure 7. Figure shows the heatmap overlayed on the WSI image by FCN models in Ensemble-B configuration. (Left to Right): 3 DenseNet-121 FCN models trained on cross-validation folds: 1, 0 and 2 respectively and Ensemble-B, (Patch Size: 1024x1024, Sampling Stride: 512 pixels). It can be seen from the heatmaps that the models struggled (relatively high posterior probability values at non-tumour regions) at extended regions from tissue boundaries. Generated using Matplotlib 3.1.1 (https://matplotlib.org/).

Table 5. Metastases type distribution in CAMELYON17 train set and validation set. The numbers indicate the number of WSI images in each metasis type.

Set	Negative	ITC	Micro	Macro	Total
Train set	100	26	35	44	215
Validation set	98	35	64	88	285

Table 6. List of features extracted for the purpose of predicting lymph node metastases type. Features were extracted after thresholding tumour probability heatmaps. For feature numbers 5, 6, 7, 8 and 9 the following statistics were computed- maximum, mean, variance, skewness, and kurtosis.

No.	Feature description	Threshold (p)
1	Largest tumour region's major axis length	p=0.9 & p=0.5
2	Largest tumour region's area	p=0.5
3	Ratio of tumour region to tissue region	p=0.9
4	Count of non-zero pixels	p=0.9
5	Tumour regions area	p=0.9
6	Tumour regions perimeter	p=0.9
7	Tumour regions eccentricity	p=0.9
8	Tumour regions extent	p=0.9
9	Tumour regions solidity	p=0.9
10	Mean of all region's mean confidence probability	p=0.9
11	Number of connected regions	p=0.9

remaining 57 patients as a validation set. The split ensured that the patients in the train set had at least one WSI image with pixel-level annotation. Table 5 shows the distribution of WSI images in terms of metastases type between train and validation sets; the proposed split strategy ensured that the distribution of metastases type between the two splits was similar.

1.2.3 Performance of Random Forest classifier without data balancing

The tumor probability heatmaps for all the 500 WSI images were generated using Ensemble-A configuration (section 1.1.3), and from the heatmaps, all the features listed in Table 6 were extracted. Post generation of features, the training set was cleaned by removing some of the outlier points. The outliers were detected based on threshold-based heuristics like the presence of significantly large tumor false-positive regions in negative cases etc. For the purpose of classifier selection, feature elimination, and hyper-parameter tuning, the classifiers were initially trained on the train set (n=215) and later validated on the held-out validation set (n=285). Experimentation on various classifiers showed that the optimal performance in terms of classification accuracy (90.18%) and Cohen's kappa score (0.9164) on the held-out validation set was achieved with Random Forest classifier with 100 trees. From Table 5, it can be observed that the data distribution was highly class imbalanced, with negative cases being the majority class and ITC cases being the minority class. This lead to misclassifications between ITC and negative cases, as evident in the confusion matrix.

Further experimentation was performed by training another Random Forest classifier on the complete training set (n=500) in order to maximize the utilization of training points. The five-fold cross-validation showed an average accuracy score of 90%, and its performance was similar to the model trained on the train set (n=215). The above two trained models are referred to as RF-PI and RF-CI (Random Forest classifiers trained on the partial and complete training set with imbalanced class data, respectively).

1.2.4 Performance of Random Forest classifier after data balancing

The train set (n=215) split and the complete training set (n=500) were separately balanced using the SMOTETomek algorithm and two Random Forest classifiers were trained using these two balanced datasets. The two trained models are referred to as RF-PB and RF-CB (Random Forest classifiers trained on Partial and Complete training set, which are Balanced data, respectively). Table 7 provides the results of the validation study performed on all four models. It was observed that post data balancing of the training dataset, the 5-fold cross-validation accuracy scores improved by a margin of 5%.

Table 7. The table provides the validation results of the four Random Forest classifiers, each trained on different subsets of the training data. Note: For the models RF-PI and RF-PB, held-out validation existed, whereas, for the other two models, it was not available as it was trained on the entire training set, and hence N.A (not applicable) is mentioned in the table. For all the models, 5-fold cross-validation accuracy was estimated on their respective training sets. These values are provided as mean (standard deviation).

	Accuracy (%)	
**Classifier**	**5-fold CV**	**Validation set**
RF-PI	87 (0.06)	90.18
RF-PB	92 (0.03)	87.02
RF-CI	89.89 (0.03)	N.A
RF-CB	94.83 (0.02)	N.A

Table 8. Segmentation results on the held-out validation set (n=25) of DigestPath dataset.

**Model**	**Dice**
DeepLabV3Plus	0.81
DenseNet-121 FCN	0.84
Inception-ResNet-V2 FCN	0.84
Ensemble	0.86

1.3 Tumour segmentation analysis on DigestPath dataset

In this section, details specific to the training and inference strategies on the DigestPath dataset are presented. Out of 660 tissue images from the DigestPath training set, 635 images were used for training, and the remaining images were used as a held-out validation set (n=25) for model selection and hyperparameter tuning. The training set (n=635) was split further into three-fold cross-validation sets; each set of data was used to train the individual models in the ensemble. A total of 80,000 patches from the entire training data were extracted, and each model was trained on a patch size of 256x256 and a batch size of 32. The model inference procedure involved extraction of patches of size 256x256 with 50% overlap between adjacent patches in batches of 32. In order to generate the binary segmentation map, the predicted tumor probability map was thresholded at 0.5. Figure 8 illustrates an example of the segmentation map generated by the proposed ensemble model. The trained models were tested on a held-out validation set (n=25), and the corresponding results are tabulated in Table 8. The uncertainty estimation in our framework allows us to generate confidence bounds. Confidence intervals basically correspond to the scale of uncertainty on an overall overlap between ground truth and prediction. Based on this, we observed an average of ± 0.014 variation in dice score on our held-out dataset.

1.4 Tumour segmentation analysis on PAIP dataset

In this section, details specific to the training and inference strategies on the PAIP dataset are presented. The tissue mask generation incorporated the post-processing step of closing morphological operation with a kernel size of 21, followed by an opening operation with a kernel size of 5, and a final dilation operation with a kernel size of 20. The training data set (n=50) was split into five-fold cross-validation and out of these five-folds, only three of them were used for training the models of the ensemble. The data was split into five-folds as opposed to three-folds to ensure that each training set had at least 40 samples. A total of 200,000 patches from the entire training dataset were extracted with equal contributions from each training sample. The models were trained with a patch size of 256 and a batch size of 32. Training was stopped when the validation loss between epochs started to increase. Depending on the model and cross-validation fold, the number of epochs it took to reach that stage was around 20 epochs.

The model inference procedure involved extraction of non-overlapping patches of size 1024x1024 in batches of 16. For the generation of segmentation maps, the generated tumor probability maps were thresholded at 0.5. The threshold was decided based on the experimental analysis for a range of threshold values on the validation set (n=10). The optimal threshold value was found to be 0.5. It was observed that lower thresholds resulted in false positives in samples and higher thresholds led to under-segmentation. Figure 9 illustrates an example of the segmentation map generated by the proposed ensemble configuration. The performance of the trained models on the validation set (n=10) released by the challenge organizers is tabulated in Table 9.

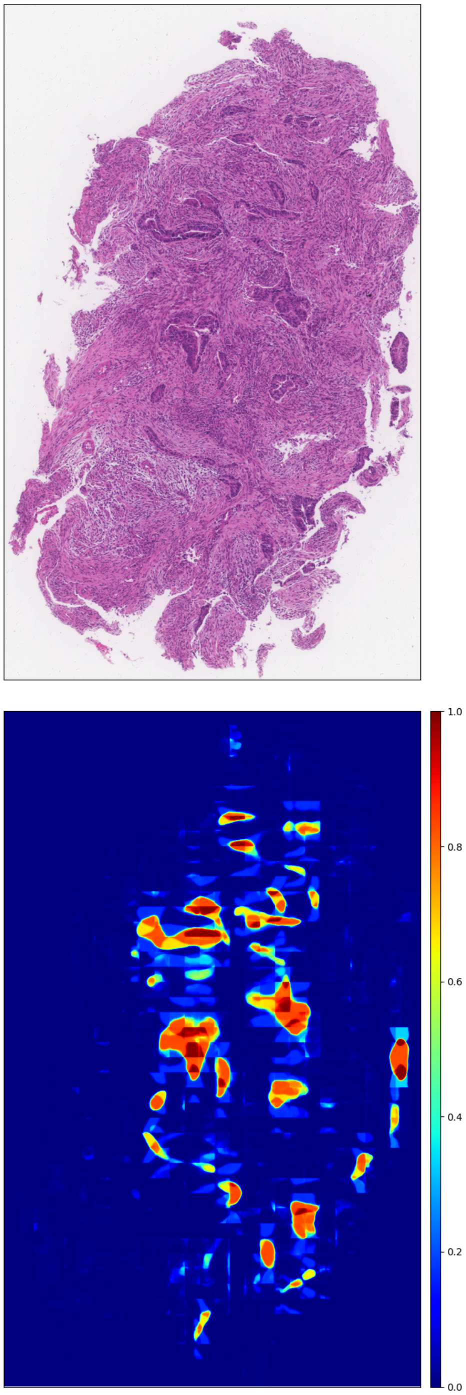

Figure 8. An illustration of the segmentation results on a sample from the DigestPath dataset. (Clockwise from top left) WSI image of H&E stained colon cancer tissue; Pathologist annotated ground truth of the tumor overlayed on the WSI image (green region indicates the tumor); Segmentation map overlayed on the WSI image (probability map thresholded at 0.5); Heatmap of Tumour probability. The detected false positives are circled (d). Generated using Matplotlib 3.1.1 (https://matplotlib.org/).

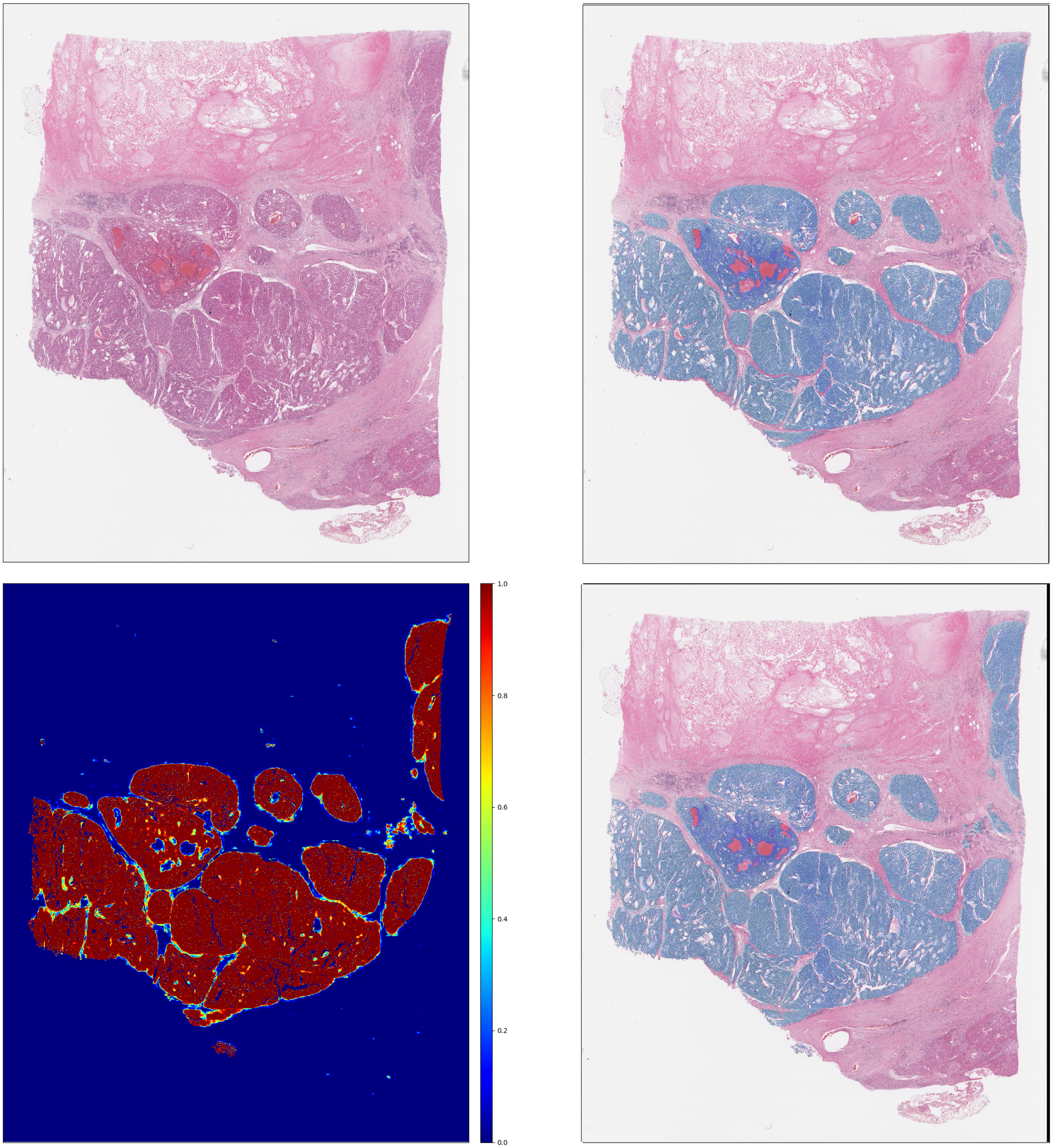

Figure 9. An illustration of the segmentation results on a sample from the PAIP dataset. (Clockwise from top left) WSI image of H&E stained liver cancer tissue; Pathologist annotated ground truth of the tumor overlayed on WSI image (blue region indicates the tumor); Segmentation map overlayed on WSI image (probability map thresholded at 0.5); Heatmap of Tumour probability. The detected false positives are circled (d). Generated using Matplotlib 3.1.1 (https://matplotlib.org/).

Table 9. Segmentation results on the validation set (n=10) of PAIP dataset.

Model	Jaccard Score
DeepLabV3Plus	0.681
Inception-ResNet-V2 FCN	0.685
DenseNet-121 FCN	0.679
Ensemble	0.701

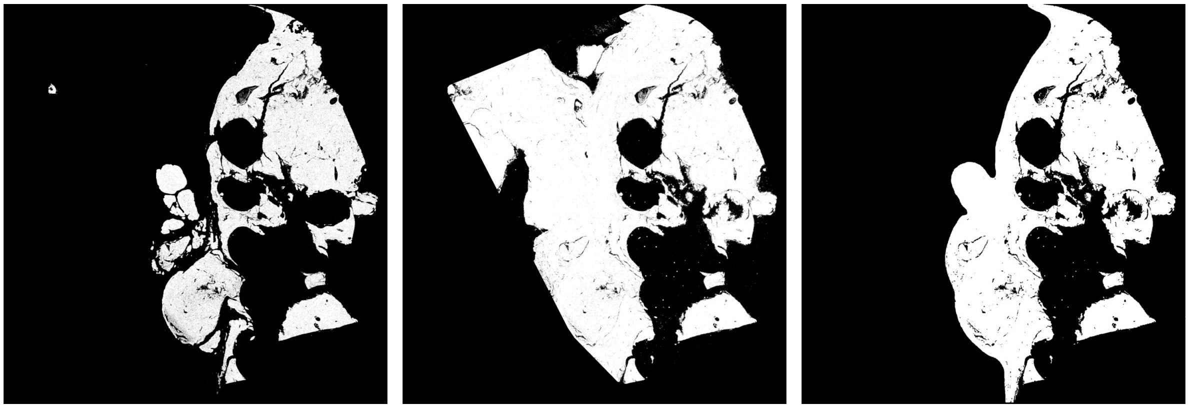

Figure 10. (Left to Right) Viable tumour prediction; Whole tumour prediction; Pathologist annotated whole tumour ground truth

1.5 Viable tumour burden analysis on PAIP dataset

Fig. 10 shows the results obtained using the proposed methodology. In (a) the predicted whole tumor segmentation was similar to pathologist provided ground truth of the whole tumor region and most of the samples in the dataset fell into this category. The proposed methodology for the whole tumor region failed in the following cases where - (b) the predicted viable tumor regions were scattered into small discrete disjoint regions which were distant from the most prominent viable tumor region and (c) the whole tumor region was larger than the convex hull of the viable tumor region.

1.6 Uncertainty analysis

In this section, we demonstrate the interpretation of the proposed uncertainty analysis. Fig. 11 and 12 provides an illustration of aleatoric and epistemic uncertainty analysis on a held-out test case from the DigestPath and CAMELYON dataset respectively. The proposed patch-based method for aleatoric uncertainty estimated high uncertainty values inside tumor regions because of prevalent loss of neighboring context information at patch borders; hence aleatoric uncertainty estimation necessitates the analysis to be conducted on a larger contiguous region of a WSI image. The proposed uncertainty analysis was done with a patch size of 256×256 because of computational constraints. In Fig. 12, (4)(d) illustrates epistemic uncertainty maps, where the uncertain regions corresponded to the boundary surrounding the tumor tissue. Figure 12 (5)(d) illustrates the map of combined uncertainties (average of aleatoric uncertainties for all the three models along with epistemic uncertainty across the three models). Figure 12 (4,5)(c), indicates that the ensemble prediction reduced the number of false positives, thereby increasing the overall Dice score to 0.94, which is about 0.04-0.06 improvement in Dice score when compared to the individual models.

1.6.1 CAMELYON uncertainty maps

Figure 12 provides an illustration of aleatoric and epistemic uncertainty analysis on a held-out test case from the CAMELYON dataset.

2 Open source contribution

An open-source application[3] on top of the proposed segmentation pipeline was developed and released. The application (Figure 13) can load WSI images, run the segmentation algorithm, and calculate the uncertainties. The software is modular making it easy for researchers to easily add their own segmentation pipelines or extend its functionality.

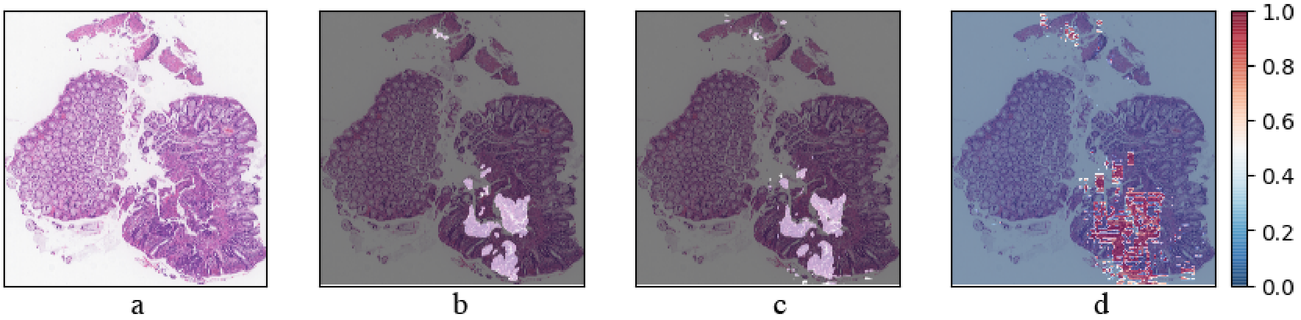

Figure 11. (a) Colon cancer tissue sample from DigestPath dataset, (b) Pathologist annotation of tumour overlayed on WSI image, (c) Tumour probability heatmaps overlayed on the WSI image and, (d) Aleatoric uncertainty maps. Generated using Matplotlib 3.1.1 (https://matplotlib.org/).

an API with which researchers can utilize the segmentation pipeline within their applications. Conversely, the application's modular structure allows researchers to test their segmentation pipeline with the application's GUI as well. The slide viewer was built using OpenSlide[4] and OpenSeadragon[5].

References

1. Abadi, M. *et al.* Tensorflow: Large-scale machine learning on heterogeneous distributed systems. *arXiv preprint arXiv:1603.04467* (2016).

2. Fleiss, J. L. & Cohen, J. The equivalence of weighted kappa and the intraclass correlation coefficient as measures of reliability. *Educ. psychological measurement* **33**, 613–619 (1973).

3. Rajkumar, H., Kori, A. & Khened, M. Digipathai (2021). Available at https://github.com/haranrk/DigiPathAI.

4. Goode, A., Gilbert, B., Harkes, J., Jukic, D. & Satyanarayanan, M. Openslide: A vendor-neutral software foundation for digital pathology. *J. pathology informatics* **4** (2013).

5. Vandecreme, A. *et al.* Openseadragon.

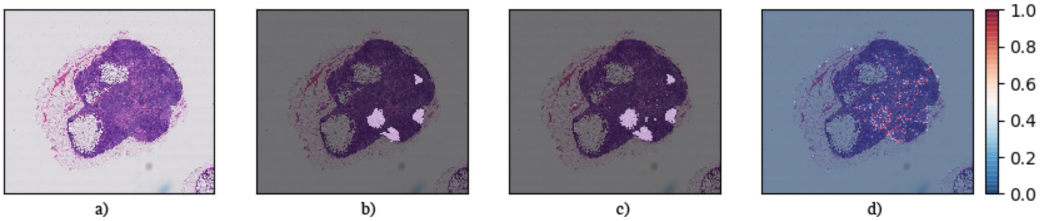

(a) (1) DenseNet-121 FCN model predictions with aleatoric uncertainty (Dice = 0.91).

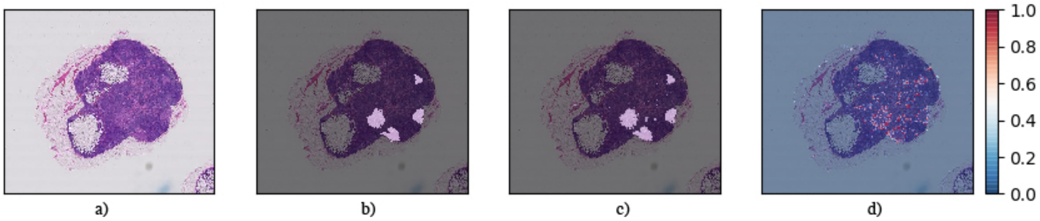

(b) (2) Inception-ResNet-V2 FCN model predictions with aleatoric uncertainty (Dice = 0.89)

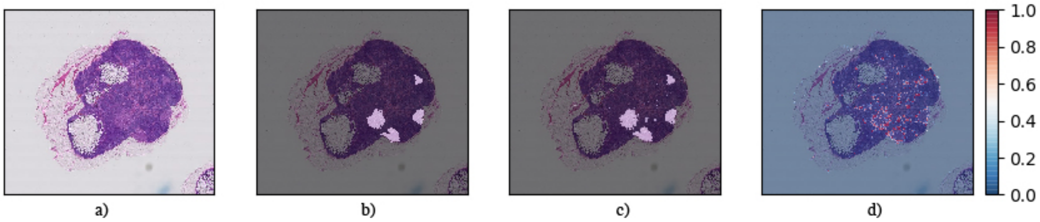

(c) (3) DeepLabv3Plus model predictions with aleatoric uncertainty (Dice = 0.88).

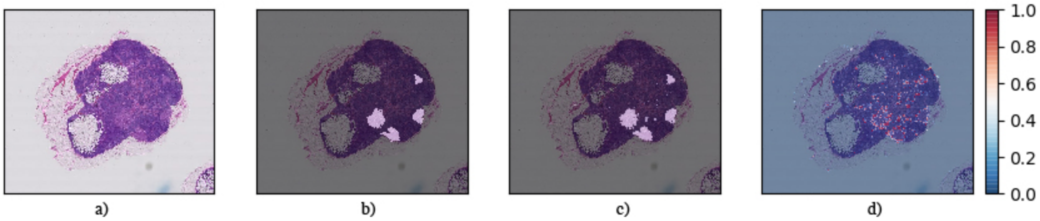

(d) (4) Ensemble model predictions with epistemic uncertainty (Dice = 0.94).

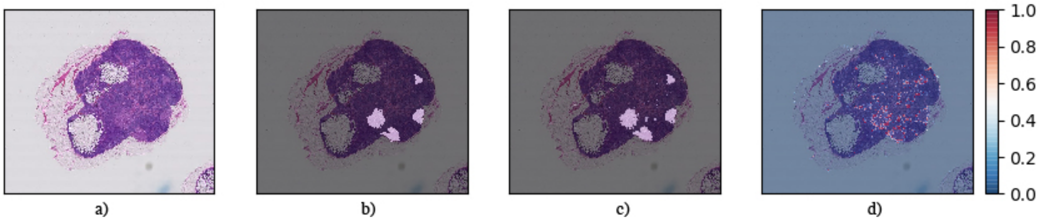

(e) (5) Ensemble model predictions with combined uncertainty (Dice = 0.94).

Figure 12. In the figure for 1-5 (a) WSI image of H&E stained lymph node section from CAMELYON dataset, (b) Pathologist annotation of tumour overlayed on WSI image, (c) Tumour probability heatmaps overlayed on the WSI image and, (d) Corresponding uncertainty analysis maps. Generated using Matplotlib 3.1.1 (https://matplotlib.org/).

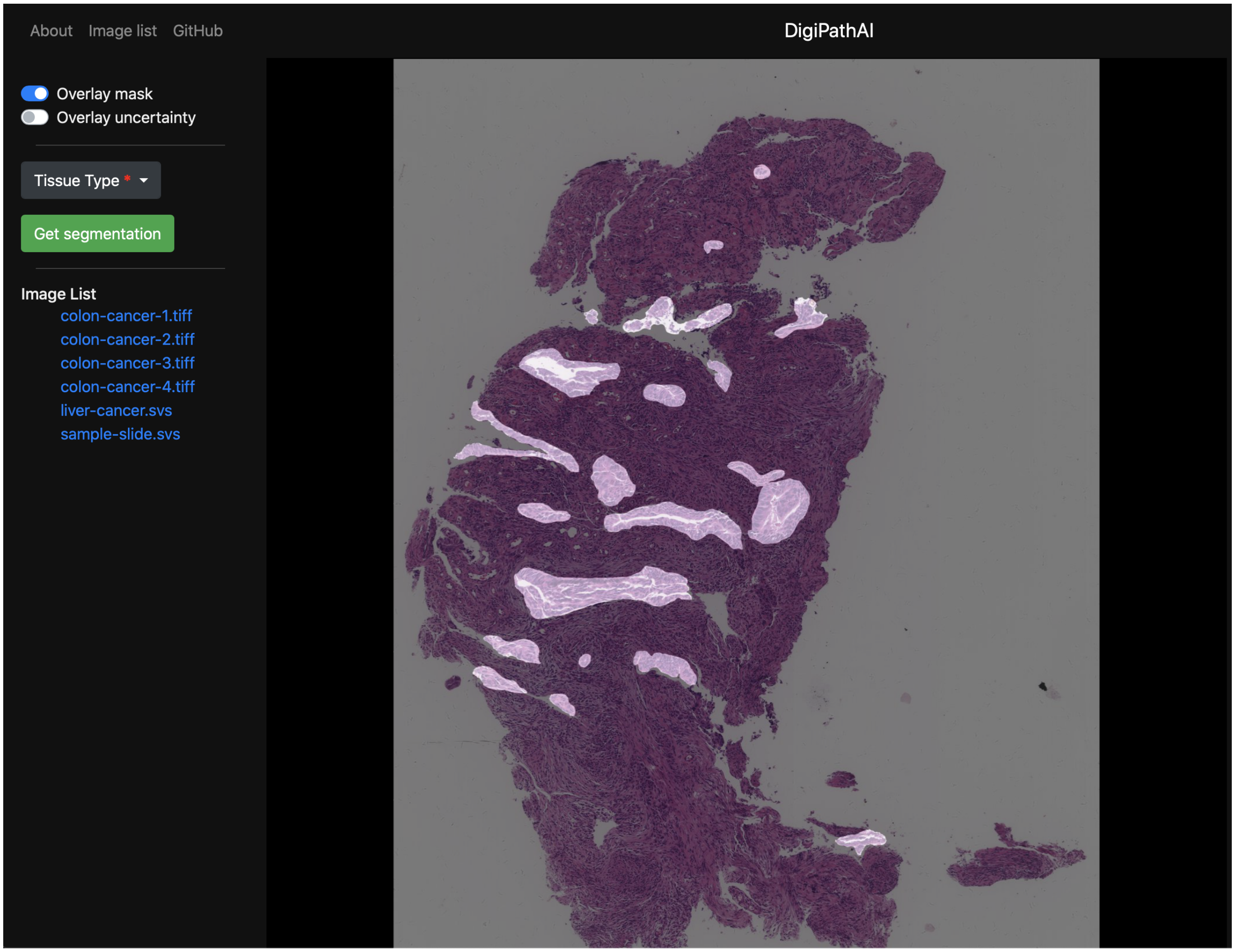

Figure 13. User interface of the WSI image analysis software.
